# Impact of maintaining serum potassium concentration ≥ 3.6mEq/L versus ≥ 4.5mEq/L for 120 hours after isolated coronary artery bypass graft surgery on incidence of new onset atrial fibrillation: Protocol for a randomized non-inferiority trial

**DOI:** 10.1371/journal.pone.0296525

**Published:** 2024-03-13

**Authors:** Niall G. Campbell, Elizabeth Allen, Richard Evans, Zahra Jamal, Charles Opondo, Julie Sanders, Joanna Sturgess, Hugh E. Montgomery, Diana Elbourne, Benjamin O’Brien

**Affiliations:** 1 Faculty of Biology, Division of Cardiovascular Sciences, School of Medical Sciences, Medicine and Health, University of Manchester, Manchester Academic Health Science Centre, Manchester, United Kingdom; 2 Manchester Heart Institute, Manchester University Foundation NHS Trust, Manchester, United Kingdom; 3 Clinical Trials Unit, London School of Hygiene and Tropical Medicine, London, United Kingdom; 4 William Harvey Research Institute, Queen Mary University of London, London, United Kingdom; 5 Division of Medicine and Institute for Sport, Exercise and Health, University College London, London, United Kingdom; 6 Department of Cardiac Anesthesiology and Intensive Care Medicine, Deutsches Herzzentrum der Charité, Berlin, Germany; 7 Department of Perioperative Medicine, St Bartholomew’s Hospital, Barts Health NHS Trust, London, United Kingdom; 8 Outcomes Research Consortium, Cleveland, Ohio, United States of America; PLOS: Public Library of Science, UNITED KINGDOM

## Abstract

**Background:**

Atrial Fibrillation After Cardiac Surgery (AFACS) occurs in about one in three patients following Coronary Artery Bypass Grafting (CABG). It is associated with increased short- and long-term morbidity, mortality and costs. To reduce AFACS incidence, efforts are often made to maintain serum potassium in the high-normal range (≥ 4.5mEq/L). However, there is no evidence that this strategy is efficacious. Furthermore, the approach is costly, often unpleasant for patients, and risks causing harm. We describe the protocol of a planned randomized non-inferiority trial to investigate the impact of intervening to maintain serum potassium ≥ 3.6 mEq/L vs ≥ 4.5 mEq/L on incidence of new-onset AFACS after isolated elective CABG.

**Methods:**

Patients undergoing isolated CABG at sites in the UK and Germany will be recruited, randomized 1:1 and stratified by site to protocols maintaining serum potassium at either ≥ 3.6 mEq/L or ≥ 4.5 mEq/L. Participants will not be blind to treatment allocation. The primary endpoint is AFACS, defined as an episode of atrial fibrillation, flutter or tachycardia lasting ≥ 30 seconds until hour 120 after surgery, which is both clinically detected and electrocardiographically confirmed.

Assuming a 35% incidence of AFACS in the ‘tight control group’, and allowing for a 10% loss to follow-up, 1684 participants are required to provide 90% certainty that the upper limit of a one-sided 97.5% confidence interval (CI) will exclude a > 10% difference in favour of tight potassium control. Secondary endpoints include mortality, use of hospital resources and incidence of dysrhythmias not meeting the primary endpoint (detected using continuous heart rhythm monitoring).

**Discussion:**

The Tight K Trial will assess whether a protocol to maintain serum potassium ≥ 3.6 mEq/L is non inferior to maintaining serum potassium ≥ 4.5 mEq/L in preventing new-onset AFACS after isolated CABG.

**Trial registration:**

ClinicalTrials.gov Identifier: NCT04053816. Registered on 13 August 2019. Last update 7 January 2021.

## Introduction

At least one in three patients is affected by atrial fibrillation after cardiac surgery (AFACS). It occurs most commonly on postoperative day two or three whilst most episodes occur within the first five postoperative days [1–3]. AFACS occurrence is associated with increases in morbidity, long-term risk of stroke [4], short and long-term mortality [2,5–7], intensive care unit (ICU) and hospital stay [8,9] and cost of care [8,10]. These associations persist after adjustment for potential confounding factors, implying a causal relationship [11]. Age is a dominant risk factor for all forms of AF and as the surgical population ages, the incidence, prevalence and healthcare impacts of AFACS are expected to increase [12].

Many of the risk factors for AFACS are the same as those for atrial fibrillation (AF) as a primary diagnosis in the general population [1,2,8]. Because many (such as age) are not modifiable, it is not known to what extent AFACS can be prevented [13]. A number of preventative strategies have been utilized but, overall, evidence to support them remains limited [14].

Potassium plays an important role in cardiac electrophysiology [15]. Serum potassium concentrations ([K+]) appear marginally lower in those suffering atrial dysrhythmias in non-surgical cohorts [16] and [K+] is commonly low following cardiac surgery [17]. It is therefore ’routine practice’ in many centres across the world to try to prevent AFACS by maintaining [K+] in the ’high-normal’ range (≥ 4.5 mEq/L), as opposed to just intervening if potassium drops below its lower ’normal’ threshold (< 3.6 mEq/L) [18,19]). However, many centres do not undertake this practice and there is large regional variation, suggesting that there is equipoise as to whether this strategy is effective [18,19].

Furthermore, potassium supplementation is not a benign intervention. Routine central venous potassium administration in the early post-operative period, when oral supplementation is not possible, is problematic. Intravenous (IV) potassium replacement requires additional fluid administration; central venous catheters which are left *in situ* for the sole purpose of potassium replacement increase infection risk; and rapid infusion can prove fatal [20]. Although individual doses of IV potassium are cheap, the annual costs of intravenous potassium exceed those for other drugs in many cardiothoracic units due to the quantities administered [21]. Nursing time (e.g. for drug checks and administration which often include more than one staff member) will further add to this cost and could limit other nursing activity. Gastrointestinal side effects, which are poorly tolerated by patients, are common after oral potassium supplementation. The benefits of such practice remain unproven.

Thus, routine maintenance of serum [K+] ≥ 4.5 mEq/L is a costly and potentially hazardous intervention, the efficacy of which remains to be proven in a trial with appropriate methodology and power. In a pilot study for this trial, we demonstrated that it was feasible to recruit and randomize patients to two different potassium supplementation protocols targeting [K+] ≥ 4.5 mEq/L (‘tight control’) and ≥ 3.6 mEq/L (‘relaxed control’) [22]. In the tight control group, 80 out of 81 patients (98.8%) had at least one measurement below 4.5 mEq/L and required potassium supplementation (unpublished data). In both arms, 45.5% of all [K+] measurements were < 4.5mEq/L at some point within the first 5 postoperative days. The pilot study demonstrated that it was possible to implement the different protocols with statistically significant differences in serum [K+] between the two arms. Fewer doses of potassium were given in the relaxed control group; a median of one supplementation was given in the relaxed control group whereas the median number of supplementations in the tight control group was six.

Based on this demonstration of feasibility, we will perform the first appropriately powered non-inferiority multicentre randomized trial to compare the incidence of new onset AFACS with ’relaxed’ vs ‘tight’ potassium supplementation. The findings will have important consequences for patients and clinicians, regardless of whether or not less aggressive potassium supplementation is found to be non-inferior for the prevention of AFACS.

## Methods

### Aim

The aim is to determine whether a strategy of maintaining serum [K+] ≥ 3.6 mEq/L (‘relaxed’ control) is non-inferior to usual treatment (target [K+] ≥ 4.5 mEq/L: ‘tight’ control) in preventing new onset AFACS after isolated coronary artery bypass graft (CABG) surgery.

### Hypothesis

New onset AFACS will be no more common (based on a non-inferiority margin of 10%) after CABG surgery when efforts are made to maintain serum [K+] ≥ PG/16/15/320503.6 mEq/L as opposed to ≥ 4.5 mEq/L.

### Setting

Twenty-three cardiac surgical centres in the United Kingdom and Germany (see Supplementary Material for a list of recruiting centres).

### Participants

A total of 1684 participants, with approximately 842 in each trial arm.

### Trial population

Adults (aged ≥ 18 years) scheduled to have elective isolated CABG surgery and both capable and willing to provide informed consent. Isolated CABG surgery is defined as CABG surgery without any additional cardiac or vascular procedure during the same operation. The harvesting of veins or arteries (e.g. internal mammary artery or radial artery) for the purposes of grafting is part of an isolated CABG procedure and not a barrier to inclusion.

Excluded will be those with, or with a history of: Atrial Fibrillation, Atrial Flutter and/or Atrial Tachyarrhythmia, or pre-operative high-degree atrioventricular (AV) block *(defined as* Mobitz type 2 second degree AV block *or* complete heart block); with a pre-operative serum [K^+^] greater than 5.5 mEq/L; with dialysis-dependent end-stage renal failure; or those who are current or previous users of medication for the purposes of cardiac rhythm management. Also excluded will be those patients enrolled in another clinical study assessing post-operative interventions aimed at modifying cardiac rhythm abnormalities. Co-enrolment with observational studies will be permitted, and for other interventional trials will be assessed by the Trial Management Group (TMG) on a case-by-case basis.

### Power calculations and sample size determination

The sample size calculation is based on a 35% incidence of new onset AFACS, a figure derived from previous large studies and is at the lower end of the published data [1,8,23]. In the pilot study for this trial, which used a similar protocol, new onset AFACS incidence was 36.9% (95% confidence interval 29.1–44.9).

The co-applicants (from diverse backgrounds in cardiac peri-operative medicine, intensive care medicine, cardiovascular nursing, cardiac surgery, cardiology, statistics, clinical trial management with patient and public involvement) reached consensus that a clinically relevant non-inferiority margin is 10%. Assuming a 35% and 37% AFACS incidence in the tight and relaxed groups respectively, 1514 participants are required to be 90% certain that the upper limit of a one-sided 97.5% CI (or equivalently a 95% two-sided CI) will exclude a difference in favour of tight potassium control of more than 10%. Allowing for a 10% loss to follow-up means that 1684 participants should be recruited.

**Fig 1:**
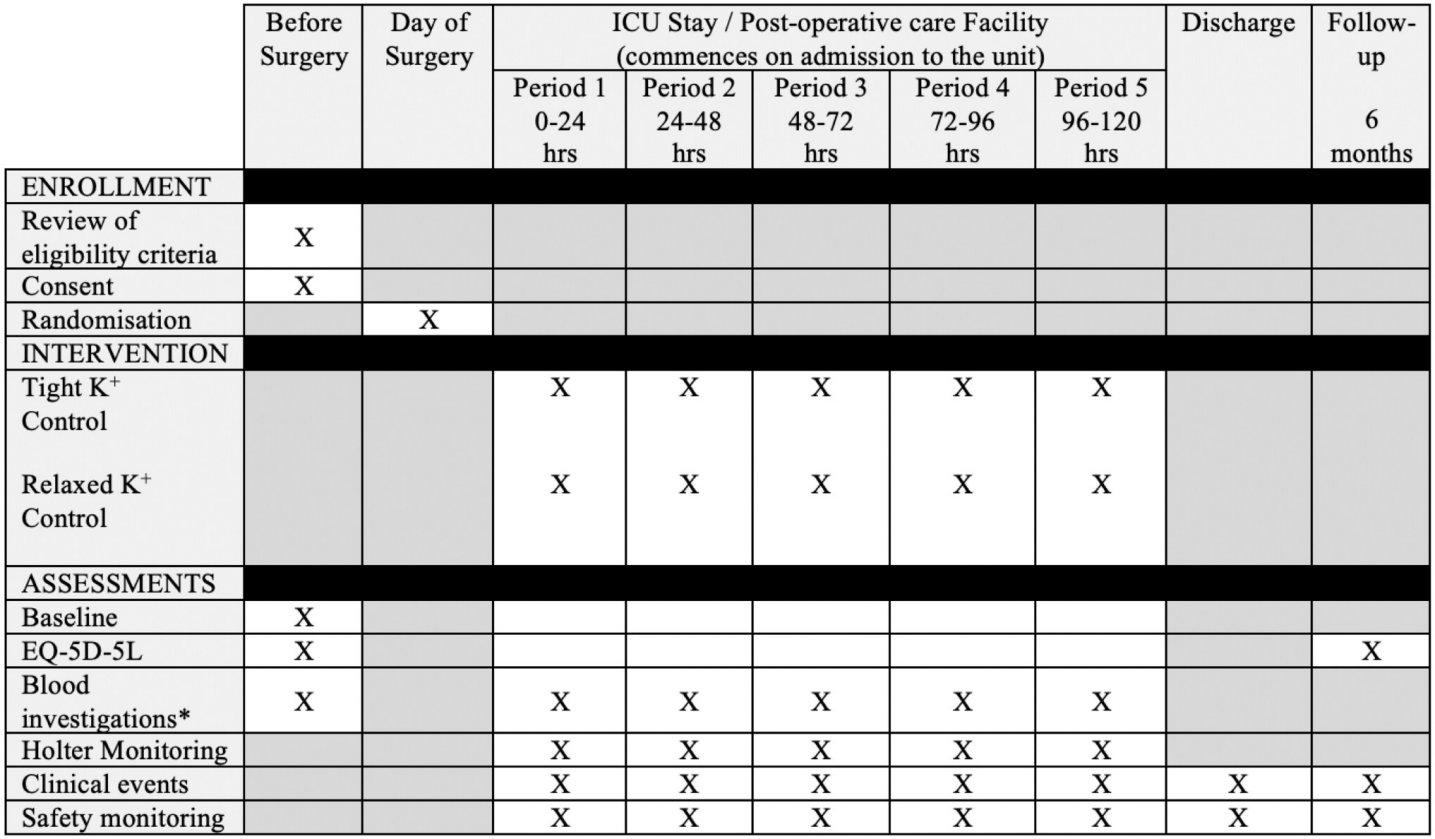
SPIRIT schedule of trial enrolment, interventions and assessments [24]. * Data recorded, but collected as part of standard care.

The SPIRIT schedule of trial enrolment, interventions and assessments is shown in Fig 1.

### Informed consent procedure

Eligible participants will be given a copy of the patient information sheet (PIS) at a pre-operative hospital appointment or upon hospital admission prior to surgery, at which time a delegated member of the research team will be available to discuss the trial and to answer patient questions. Prior to their scheduled hospital appointment, research staff will be allowed to approach patients via post, telephone or email to discuss the study.

It is recommended that patients will be allowed 24 hours to consider whether or not to take part in the study. However, if patients are willing, written consent will be obtained and is permitted at any time prior to surgery. The Consent Form is shown in the Appendix.

### Randomization

Overall, 1684 consented participants will be allocated in a 1:1 ratio using an online tool (https://sealedenvelope.com/) to target either tight (K^+^ ≥ 4.5mMol/L) or relaxed (K^+^ ≥ 3.6mMol/l) potassium control. Patients will be randomized and allocated to their treatment groups on the day of surgery by the local research team. The allocation will be stratified by site.

### Trial treatment period

**Fig 2:**
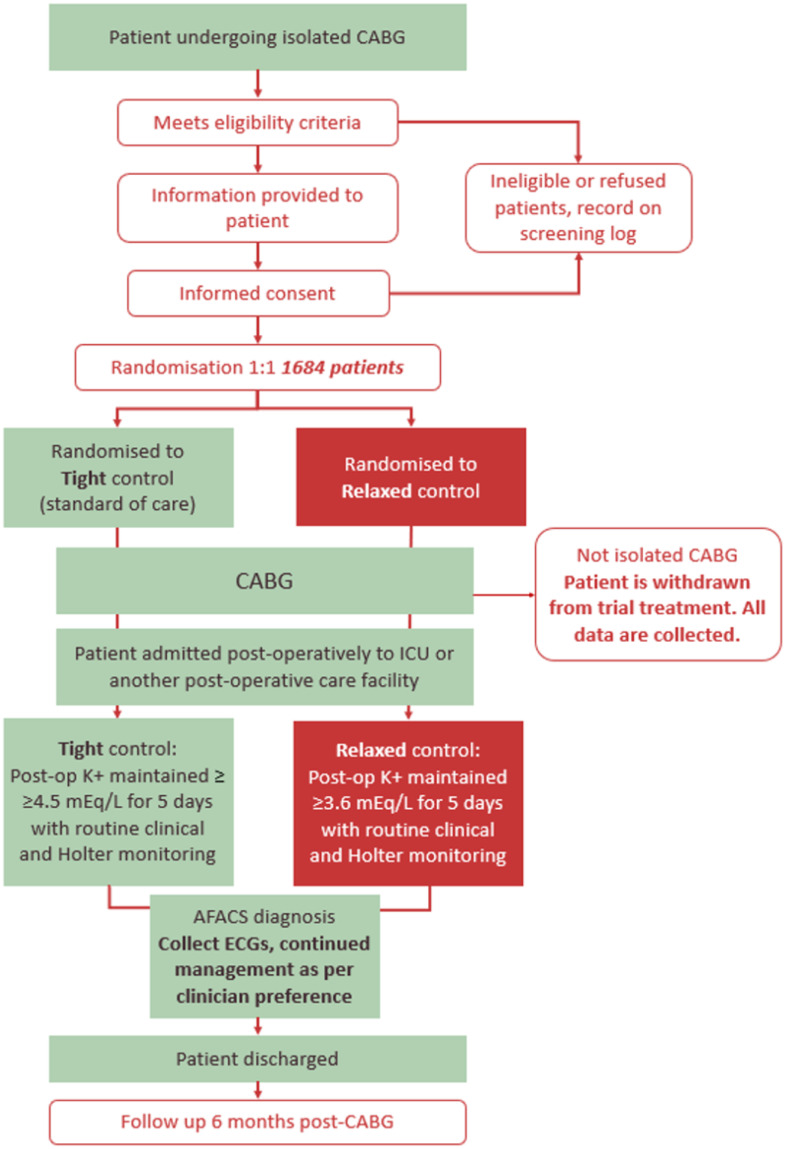
Flowchart of the study protocol. CABG: Coronary artery bypass grafting; AFACS: Atrial fibrillation after cardiac surgery; K+: Serum potassium level.

A flowchart of the trial treatment intervention process is shown in Fig 2. The trial treatment intervention period will commence when the patient is admitted to ICU or any other post-operative care facility after surgery, according to local practice. It will end 120 hours (5 days) after that time point, or with occurrence of a clinically identified episode of AFACS (see following)—whichever occurs first. Patients for whom surgery took place, but who did not undergo an isolated CABG, will be treated according to local standard of care; these patients will be followed up as normal with all data collected as per the full trial treatment period, including Holter monitor analysis and completion of the 6-month follow-up visit.

### Interventions

Participants randomized to ’Relaxed Control’ will receive K^+^ supplementation only if their serum [K^+^] drops below 3.6mEq/L. Patients randomized to ’Tight Control’ will receive K^+^ supplementation only if their serum [K^+^] falls below 4.5 mEq/L (current practice). Once a participant has a site-reported episode of AFACS, there will be no restriction on potassium supplementation and the participant should be treated according to local practice. Inpatient data collection will continue until hour 120 after admission to ICU/post-operative care facility or discharge from hospital, whichever is shorter.

The frequency of [K+] monitoring will be according to local protocols, clinician/nursing staff preference and clinical need. The route of administration for all potassium replacement will be according to clinician preference and according to existing standardized protocols, and may include intravenous (IV) or oral potassium formulation, administration of potassium-rich nasogastric feeding regimens, recommending the consumption of potassium-rich foods or avoidance of potassium losing drugs. Blinding patients and clinical staff to the treatment allocation post randomisation is not feasible.

All other treatments, including IV magnesium, beta-blockers or anti-dysrhythmic agents for the management of cardiac rhythm, will be given according to standard clinical care and clinician preference.

An external heart rhythm monitor (‘Holter’) will be applied just before (or as soon as possible after) the patient enters the ICU / post-operative care facility and the time or application will be documented. Monitoring will continue until hour 120 (5 days) or discharge from hospital, whichever is sooner whether or not site-reported AFACS occurs. However, if the Holter monitor is removed after a site-reported period of AFACS (e.g. for cardioversion), it need not be replaced.

### Data

At recruitment, demographic data, medical history and relevant medication will be documented, as will CHA_2_D_2_VASC score (a risk score for thromboembolic risk with atrial fibrillation) [25] and EUROSCORE2 (a risk score for mortality following cardiac surgery) [26].

During the intervention period, data related to adverse events attributed to K+ replacement will be recorded, including gastrointestinal symptoms from oral K+ replacement, the need for temporary cardiac pacing and duration of central venous cannulation.

Additional staff time for delivering potassium will be recorded, informed by expert clinical view. Detailed information will be collected on the resource use associated with delivering each protocol, including the total number of replacement K+ interventions and the number of tests for monitoring potassium levels.

At hospital discharge, data relating to relevant medication given during hospital stay will be collated, including whether anticoagulation was commenced for AFACS. Length of ICU and hospital stay will be recorded.

Anonymised Holter data will be extracted and analysed (blind to participant allocation and after the trial treatment period has ended) by the core laboratory group based at Wythenshawe Hospital, Manchester University NHS Foundation Trust, United Kingdom. Data therefore cannot be used to guide patient management in real time. Dysrhythmia episodes (as defined above) will be identified and their number / durations will be recorded.

### Follow-up

All surviving participants will be followed up (in person, via a telephone call, email or post) 6 months after surgery (+/- 1 month), or 6 months (+/- 1 month) after randomisation if surgery did not proceed. An EQ-5D-5L (a self-assessed, health-related, quality of life) questionnaire will be completed by the patient at this time. Participants will be asked to provide information about further episodes of AFACS, other heart rhythm problems and stroke after their hospital discharge, if known.

Participants will also be followed up remotely via NHS Digital or via Public Health Scotland to identify any hospitalisations for AFACS between discharge and 6 months post-surgery.

### End of trial

The trial will conclude with the last follow-up of the last participant.

### Definitions

AFACS will be defined as an episode of atrial fibrillation, flutter or tachyarrhythmia. The minimum duration of documented uninterrupted atrial fibrillation, flutter or tachyarrhythmia required to establish the diagnosis of AFACS is at least 30 seconds, or an entire 12-lead ECG [27].

Episodes lasting <30 seconds will not be counted for the purposes of the primary or secondary endpoints.

It can be challenging to discriminate between atrial fibrillation, atrial flutter and atrial tachyarrhythmias on electrocardiographic grounds alone and the treatment in the post-operative clinical setting is typically the same. For the purposes of this study, they will thus be categorized together and referred to as AFACS.

Local guidelines will be used to diagnose any of the three contributing atrial dysrhythmias.

ECG criteria for ***atrial fibrillation*** are [28]:

1. Absolutely irregular RR intervals in the absence of complete AV block
2. No distinct P waves on the surface ECG
3. An atrial cycle length (when visible) that is usually variable and which is less than 200ms.

***Atrial flutter*** refers to a regular tachycardia with atrial rate ≥ 240 beats per minute lacking an isoelectric baseline between deflections. A characteristic ECG ‘sawtooth’ pattern in leads II, III and/or aVF is common, although continuous undulation of the atrial complex without a sawtooth appearance can sometimes be identified.

***Atrial tachycardia*** is a regular atrial rhythm with a sudden onset / offset at a constant atrial rate ≥ 100 beats per minute, with an isoelectric baseline between deflections. The P-wave morphology is different to that of sinus rhythm and the ventricular rate is usually regular.

#### Definition of Holter-Identified AFACS

Holter-Identified AFACS will be defined as an episode of atrial fibrillation, flutter or tachyarrhythmia (as defined above) lasting ≥ 30 seconds that is detected on Holter monitoring. Episodes lasting < 30 seconds will not be counted for the purposes of the secondary endpoints.

#### Definition of non-AFACS dysrhythmias

Non-AFACS supraventricular dysrhythmia of ≥ 30 seconds

Ventricular tachycardia/fibrillation (defined as more than 3 beats at ≥ 100bpm)

Mobitz type 2 block (any duration)

Complete heart block (any duration)

Other ventricular pause ≥ 3 seconds

### Outcomes

Primary outcome

- The presence of new onset AFACS that is both clinically detected and electrocardiographically confirmed (on either electrocardiogram (ECG), telemetry or Holter monitoring) until hour 120 after admission to ICU/post-operative care facility or discharge from hospital, whichever occurs first

Secondary outcomes

- The incidence of new onset AFACS detected on Holter monitor until hour 120 after initial admission to ICU/post-operative care facility or discharge from hospital, whichever occurs first
- The incidence of at least one episode of AFACS (clinically identified) or Holter-identified AFACS (where none identified clinically) until the end of hour 120 after initial admission to ICU/post-operative care facility or discharge from hospital, whichever occurs first
- The number of patients experiencing at least one episode of a non-AFACS arrhythmia, identified on Holter monitors until hour 120 after initial admission to ICU/post-operative care facility or discharge from hospital, whichever occurs first
- In-patient mortality
- 6-month mortality
- Length of stay on ICU, a coronary care unit or fast-track post-operative ward facility
- Hospital length of stay
- Costs relating to purchasing and administering potassium therapy
- Quality of life at 6 months

### Adjudication of primary outcome

The primary outcome will be independently adjudicated and validated using a hierarchy approach to validate the primary outcome by an Event Validation Committee (EVC). The aim is to address any concerns of bias in reporting the primary outcome and to ensure that when the trial results are published, the results will be considered robust.

**Fig 3:**
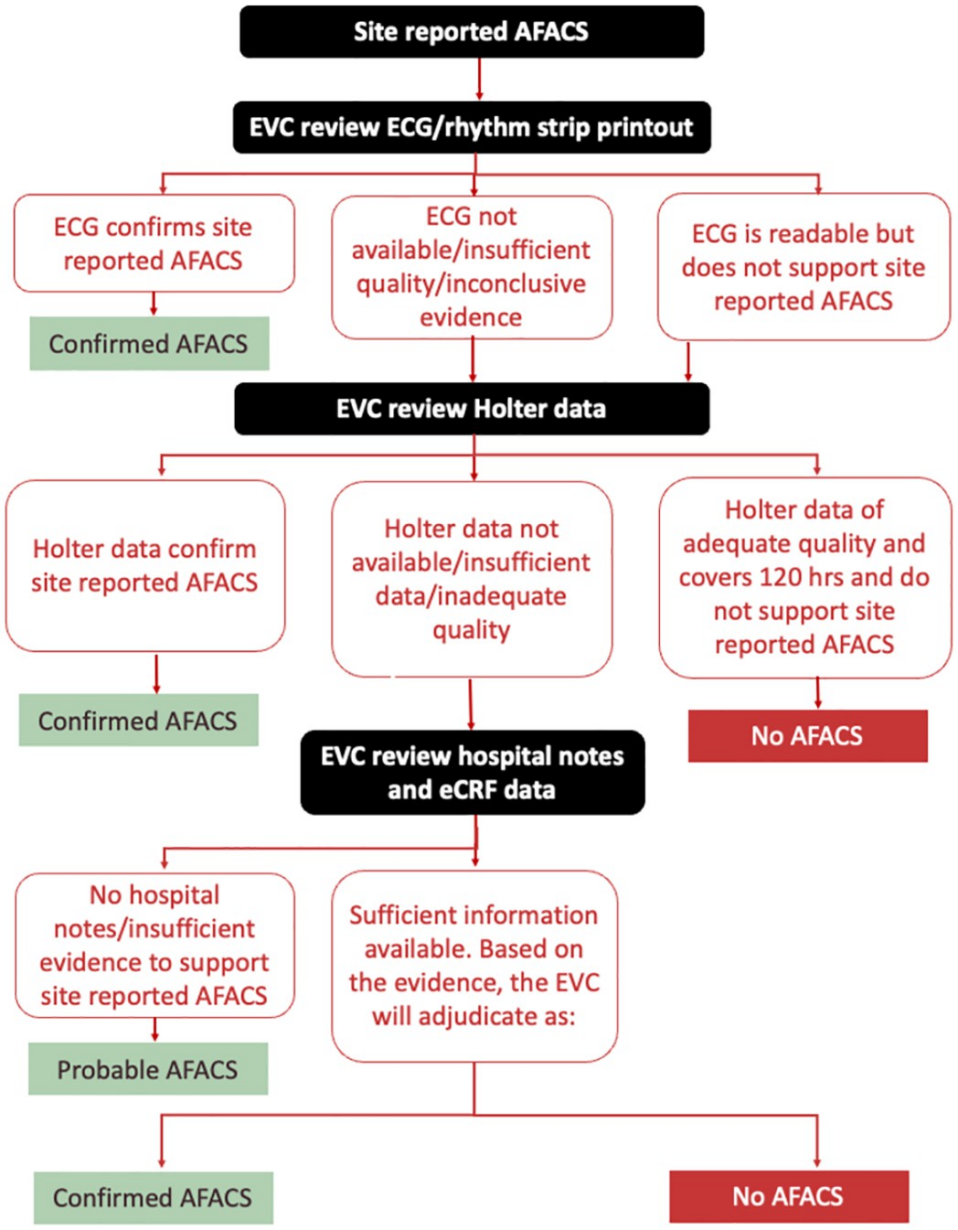
Arbitration flowchart for determining if primary endpoint was met after site reported atrial fibrillation after cardiac surgery (AFACS).

The premise of the hierarchy approach is that site-reported AFACS is adjudicated against the best-available evidence (see flowchart, Fig 3).

**Category I evidence: An ECG/telemetry/rhythm strip printout provided by the site**:

- If the ECG/rhythm strip is readable and confirms an uninterrupted episode of atrial fibrillation, atrial flutter or atrial tachyarrhythmia, the event will be classified as “Confirmed AFACS”
- If the ECG/rhythm strip is readable but there is no evidence of an uninterrupted episode of atrial fibrillation, atrial flutter or atrial tachyarrhythmia, then proceed to reviewing Category II evidence.
- If the ECG/rhythm strip is not available or not readable or if evidence of an uninterrupted episode of atrial fibrillation, atrial flutter or atrial tachyarrhythmia is inconclusive, then proceed to reviewing Category II evidence.

**Category II evidence**: **Holter monitor data collected for that participant**

- If an episode of atrial fibrillation, atrial flutter or atrial tachyarrhythmia lasting ≥ 30 seconds is recorded on the Holter monitor, then the event will be classified as “Confirmed AFACS”
- If Holter data are not available, or there is insufficient data due to gaps in the recording, or the data is of insufficient quality, then proceed to reviewing Category III evidence
- If Holter data are of adequate quality and cover the full period of 120 hours (or up to discharge if sooner), and an uninterrupted episode of atrial fibrillation, atrial flutter or atrial tachyarrhythmia lasting ≥ 30 seconds is not recorded on the Holter monitor, then the event will be classified as “No AFACS”.

**Category III evidence**: **Supporting evidence in hospital notes and in the electronic case record form (eCRF)**

- The committee will review circumstantial supporting evidence from the eCRF, such as administration of amiodarone or DC/chemical cardioversion, alongside patient notes. Based on the level of evidence available, it will decide whether the event can be classified as either “Confirmed AFACS” or “No AFACS”
- If no hospital notes are available or there is insufficient evidence to confirm or refute the presence of AFACS, the event will be classified as “Probable AFACS”

The intention is that initially Category I and Category II evidence will be sent to the EVC, and only if necessary, will category III evidence be collected from the sites and sent to the EVC at a later stage. The committee members will be blinded to treatment allocation.

### Statistical analysis

Statistical analysis will be performed by statisticians based at the London School of Hygiene and Tropical Medicine’s Clinical Trials Unit (LSHTM CTU). Outcome analysis will be blind to treatment allocation. A formal statistical analysis plan (SAP) will be published before data lock. In brief, baseline characteristics of enrolled participants will be summarized by treatment arm. Descriptive statistics for continuous variables will include mean, standard deviation, median, range and number of observations. Categorical variables will be summarized as counts and proportions. Screening, enrolment, reasons for non-enrolment, randomisation and loss to follow-up will be detailed in a CONSORT flowchart [29].

The primary and secondary efficacy outcome analysis will be carried out using all randomised participants assigned a randomisation number who underwent isolated CABG surgery, excluding patients who did not undergo isolated CABG. An analysis on a per-protocol population will also be carried out excluding participants not completing a protocol-adherent course of treatment.

All participants assigned a randomisation number will be considered for the ITT safety analysis. This will include some participants who will have been excluded from the efficacy analysis population.

The primary analysis will be unadjusted and carried out using the efficacy population.

For the primary outcome (presence of new onset AFACS up to the end of hour 120 after admission to ICU/post-operative care facility or discharge from hospital), we will use a one-sided 97.5% confidence interval approach to test for non-inferiority between the two treatments. The one-sided 97.5% CI for the between group difference point estimate will be calculated using a binomial regression model. Non-inferiority of the relaxed arm will be accepted if the upper bound of the 97.5% CI lies within the pre-specified non-inferiority margin of 10%.

For secondary outcomes, the analysis will be based on tests for superiority comparing the two arms of the trial using two-sided tests at the 5% level of significance. Logistic regression models will be used for analysis of mortality, and linear regression models will be used to analyse lengths of stay, costs and quality of life scores.

An analysis will also be performed which is adjusted for the stratification factor (site) and any other adjustment factors pre-specified in the SAP. Additional exploratory analyses will control for any baseline measures that appear to be imbalanced between arms. All subgroup analyses will be specified *a priori* in the SAP and performed using formal tests for interaction included in the statistical models and assessed for statistical significance using Likelihood ratio tests.

No datasets were analysed during the current study for this manuscript. All relevant data from this study will be made available upon study completion.

### Trial Governance

Participant data will be kept confidential and managed in accordance with the Data Protection Act, NHS Caldicott principles, The Research Governance Framework for Health and Social Care, and the conditions of Research Ethics Committee Approval.

Participants may withdraw from the trial at any time without prejudice to their future care and will undergo standard clinical care. Participants who withdraw from the trial after discharge from hospital will be followed up as per standard clinical care by the local clinical team. Participants will be encouraged to allow data that have been collected before withdrawal to be used in the analyses. However, if consent to use already collected data is also withdrawn, then these data will be discarded. There will be no further follow-up from the research team.

Barts NHS Trust, as Sponsor of this trial, will ensure that arrangements are in place to record, notify, assess, report, analyse, and manage adverse events in order to comply with the UK regulations of Medicines for Human Use (Clinical Trials) Regulations 2004. All sites involved in the trial will be expected to inform the Chief Investigator of any unexpected serious adverse events/reactions within 7 days so that appropriate safety reporting procedures can be followed by the Sponsor. The Chief Investigator will be responsible for the prompt notification of findings that could adversely affect the health of patients or impact on the conduct of the trial. Notification of confirmed expected and related SAEs will be to the Sponsor, the Research Ethics Committee and the Data and Safety Monitoring Committee (DSMC).

Relevant trial documentation will be kept for a minimum of 15 years. Electronic data will be stored in a fully audited data centre in the UK with appropriate certifications including ISO 27001:2005 (Information Security) and 9001:2008 (Quality Management).

The trial is registered with ClinicalTrials.gov, identifier: NCT04053816. We will follow the Medical Research Council Guidelines on GCP in Clinical Trials. All investigators have been trained in GCP. The study results will be reported according to the CONSORT guidelines.

The trial will be overseen by the Trial Steering Committee (TSC), including an independent chair, public-patient representatives and at least two other independent members. The TSC will meet periodically during the trial.

Local investigators will ensure that all trial data are available for trial-related monitoring, and sponsor and regulatory authority audits. The sponsor also holds the right to monitor or audit the study.

The DSMC will be independent of the investigators and of the TSC, but will report to the TSC and (via the TSC) to the Sponsor. The DSMC will consist of an independent chair, a senior statistician and at least one other senior clinician independent of the investigators. The DSMC will meet prior to the start of the trial and monitor safety on an ongoing basis. They will also monitor data for quality and completeness. This will be facilitated through the development of a trial-specific database and an adverse-event database. Given that this is a non-inferiority trial no formal interim analyses are planned. Patient data will be kept confidential and managed in accordance with the Data Protection Act (2018), NHS Caldicott principles, the Research Governance Framework for Health and Social Care, and the conditions of Research Ethics Committee Approval.

The trial will be directed by the chief investigator and a project management group which will include the trial manager, data manager and trial statistician.

At the end of the study, the data will be analysed and reported in a peer reviewed journal according to CONSORT standards [29]. The authors will ensure these data are publicly available, including to trial participants. The results will also be uploaded to the clinicaltrials.gov database. After publication, the TSC will consider applications to share the anonymized dataset with external researchers.

The SPIRIT 2013 Checklist: Recommended items to address in a clinical trial protocol and related documents is shown in the Supplementary Materials.

Trial Protocol Version 1 and Version 3 (the most recent version) are included in the Supplementary Materials.

## Discussion

Atrial arrhythmias are common after CABG. Whilst postoperative serum potassium levels have been associated with reduced AF risk after CABG [1], these data are retrospective and no benefit of a specific potassium target can be inferred. The common practice of maintaining high-normal serum [K+] is thus unproven in preventing such events. The Tight K Trial will address the efficacy of this practice in a non-inferiority, individually randomized trial.

There is wide variation in how atrial fibrillation is defined in interventional studies, and the reasons why a specific definition has been utilized is rarely adequately described [30]. In our study, we will use a ‘real world definition’ of AFACS as the primary endpoint–the dysrhythmic episode will need to be both clinically identified and subsequently confirmed by electrocardiographic documentation. The investigators have opted for an AFACS definition that is aligned with the 2020 European Society of Cardiology guidelines for the diagnosis of atrial fibrillation [27].

Holter monitoring during the interventional period may assist with this. An arbitration process will be used (Fig 3) that will adjudicate whether clinically-identified episodes of AFACS have occurred, whilst still being respectful of the real-world nature of the trial. To our knowledge, this is the first time that such a process has been deployed. This will address the possible bias of over-reporting of AFACS. There is also potential for sites to under-report AFACS. Missing a period of AFACS at a site may not be that uncommon and does not necessarily lead to bias, unless it is unbalanced between the two arms.

In routine practice, episodes of atrial fibrillation, atrial flutter and atrial tachycardia can overlap and be difficult to distinguish for healthcare staff who are not trained electrophysiologists. The three dysrhythmias also have similar clinical management pathways and risk of thromboembolism. Including these three dysrhythmias in the primary endpoint is therefore a pragmatic real-world solution to this problem. Standardisation of the definition of AFACS and other dysrhythmias are needed in future to ensure that there is consistency across patient populations in interventional studies [30].

Our pilot study helped inform the design of this definitive study. A protocol violation when either (a) potassium supplementation was administered when it should have been withheld or (b) supplementation was withheld when it should have been administered, occurred in 9.8% (283/2886) of patients [22]. To help minimise such events, patients will now wear colour coded wristbands to help staff to identify the group to which they have been allocated. In addition, in response to the critical need for all junior medical staff and all nursing staff to be aware of the trial, and to understand its protocols, being identified, materials were developed to remind staff about the study and treatment allocation groups. It also appeared that patients can play a role in reminding staff of their trial allocation group, and we shall encourage this engagement.

As an open label study, there is a hypothetical possibility of detection bias. Whilst the investigators believe this unlikely to occur in practice, independent validation of site-reported events will enhance the rigour of the trial. There is also potential for sites to under-report AFACS. Missing a period of AFACS at a site may not be that uncommon and does not necessarily lead to bias, unless it is unbalanced between the two arms.

The findings will have important consequences for patients and clinicians, regardless of whether or not potassium supplementation is found to be non-inferior for the prevention of AF after cardiac surgery. Genuine equipoise appears to exist internationally. In European institutions, 67% of caregivers practices have a protocol for maintaining high-normal serum potassium levels after cardiac surgery, whilst one in three does not [19]. The practice of maintaining high-normal serum potassium levels anecdotally appears less common in the United States.

We anticipate that our findings will be of substantial clinical relevance: if effective, potassium supplementation to a high target level could be implemented as a standard of care, and peri-operative morbidity will be reduced. If of no benefit, then an unnecessary intervention can be avoided with a potentially significant reduction in risks of administration and cost.

## Supporting information

S1 File SPIRIT 2013 checklist: Recommended items to address in a clinical trial protocol and related documents. (DOC)

S2 File A List of recruiting sites for the Tight K Trial. (DOCX)

S3 File Protocol for the Tight K Trial, Version 1 (April 2019). (PDF)

S4 File Protocol for the Tight K Trial, Version 3 (February 2023). (PDF)

## Abbreviations

AF: Atrial Fibrillation (including Atrial Flutter / Tachycardia in definition)
AFACS: Atrial Fibrillation After Cardiac Surgery (including Atrial Flutter / Tachycardia in definition)
CABG: Coronary Artery Bypass Grafting
DMSC: Data monitoring and safety committee
ECG: electrocardiogram
eCRF: Electronic Case Record Form
EVC: Event validation committee
ICU: Intensive Care Unit
IV: Intravenous
K+: Potassium
SAP: Statistical Analysis Plan
TMG: Trial Management Group
TSC: Trial Steering Committee

## References

[1] Mathew JP, Fontes ML, Tudor IC, Ramsay J, Duke P, Mazer CD, et al. A multicenter risk index for atrial fibrillation after cardiac surgery. JAMA. 2004;291: 1720–9. doi: 10.1001/jama.291.14.1720 15082699

[2] Helgadottir S, Sigurdsson MI, Ingvarsdottir IL, Arnar DO, Gudbjartsson T. Atrial fibrillation following cardiac surgery: risk analysis and long-term survival. J Cardiothorac Surg. 2012;7: 87. doi: 10.1186/1749-8090-7-87 22992266 PMC3515503

[3] Greenberg JW, Lancaster TS, Schuessler RB, Melby SJ. Postoperative atrial fibrillation following cardiac surgery: a persistent complication. Eur J Cardio-thorac. 2017;52: 665–672. doi: 10.1093/ejcts/ezx039 28369234

[4] Benedetto U, Gaudino MF, Dimagli A, Gerry S, Gray A, Lees B, et al. Postoperative Atrial Fibrillation and Long-Term Risk of Stroke After Isolated Coronary Artery Bypass Graft Surgery. Circulation. 2020;142: 1320–1329. doi: 10.1161/CIRCULATIONAHA.120.046940 33017213 PMC7845484

[5] Villareal RP, Hariharan R, Liu BC, Kar B, Lee V-V, Elayda M, et al. Postoperative atrial fibrillation and mortality after coronary artery bypass surgery. JACC. 2004;43: 742–748. doi: 10.1016/j.jacc.2003.11.023 14998610

[6] Mariscalco G, Klersy C, Zanobini M, Banach M, Ferrarese S, Borsani P, et al. Atrial fibrillation after isolated coronary surgery affects late survival. Circulation. 2008;118:1612–1618—Google Search. Circulation. 2008;118: 1612–1618. doi: 10.1161/CIRCULATIONAHA.108.777789 18824644

[7] El-Chami MF, Kilgo P, Thourani V, Lattouf OM, Delurgio DB, Guyton RA, et al. New-onset atrial fibrillation predicts long-term mortality after coronary artery bypass graft. JACC. 2010;13: 1370–1376. doi: 10.1016/j.jacc.2009.10.058 20338499

[8] Aranki SF, Shaw DP, Adams DH, Rizzo RJ, Couper GS, VanderVliet M, et al. Predictors of Atrial Fibrillation After Coronary Artery Surgery: Current Trends and Impact on Hospital Resources. Circulation. 1996;94: 390–397. doi: 10.1161/01.cir.94.3.390 8759081

[9] Sanders J, Keogh BE, der Meulen JV, Browne JP, Treasure T, Mythen MG, et al. The development of a postoperative morbidity score to assess total morbidity burden after cardiac surgery. J Clin Epidemiol. 2012;65: 423–433. doi: 10.1016/j.jclinepi.2011.11.004 22360990

[10] Zimmer J, Pezzullo J, Choucair W, Southard J, Kokkinos P, Karasik P, et al. Meta-analysis of antiarrhythmic therapy in the prevention of postoperative atrial fibrillation and the effect on hospital length of stay, costs, cerebrovascular accidents, and mortality in patients undergoing cardiac surgery. Am J Cardiol. 2003;91: 1137–1140. doi: 10.1016/s0002-9149(03)00168-1 12714166

[11] Borzak S, Tisdale JE, Amin NB, Goldberg AD, Frank D, Padhi ID, et al. Atrial fibrillation after bypass surgery: does the arrhythmia or the characteristics of the patients prolong hospital stay? Chest. 1998;113: 1489–1491. doi: 10.1378/chest.113.6.1489 9631782

[12] Schnabel RB, Sullivan LM, Levy D, Pencina MJ. Development of a risk score for atrial fibrillation (Framingham Heart Study): a community-based cohort study. Lancet. 2009;373: 739–745. doi: 10.1016/S0140-6736(09)60443-8 19249635 PMC2764235

[13] Campbell NG, O’Brien B. More pumps better? J Cardiothor Vasc An. 2020;34: 2948–2950. doi: 10.1053/j.jvca.2020.07.073 32843272

[14] Burrage PS, Low YH, Campbell NG, O’Brien B. New-Onset Atrial Fibrillation in Adult Patients After Cardiac Surgery. Curr Anesthesiol Rep. 2019;9: 174–193. doi: 10.1007/s40140-019-00321-4 31700500 PMC6837869

[15] Podrid PJ. Potassium and ventricular arrhythmias. Am J Cardiol. 1990;65: 33E–44E; discussion 52E. doi: 10.1016/0002-9149(90)90250-5 2178376

[16] Krijthe BP, Heeringa J, Kors JA, Hofman A, Franco OH, Witteman JCM, et al. Serum potassium levels and the risk of atrial fibrillation. Int J Cardiol. 2013;168: 5411–5415. doi: 10.1016/j.ijcard.2013.08.048 24012173

[17] Polderman KH, Girbes AR. Severe electrolyte disorders following cardiac surgery: a prospective controlled observational study. Crit Care. 2004;8: R459–66. doi: 10.1186/cc2973 15566592 PMC1065069

[18] O’Brien B, Burrage PS, Ngai JY, Prutkin JM, Huang C-C, Xu X, et al. Society of Cardiovascular Anesthesiologists/European Association of Cardiothoracic Anaesthetists Practice Advisory for the Management of Perioperative Atrial Fibrillation in Patients Undergoing Cardiac Surgery. J Cardiothor Vasc Anesth. 2019;33: 12–26. doi: 10.1053/j.jvca.2018.09.039 30591178

[19] Muehlschlegel JD, Burrage PS, Ngai JY, Prutkin JM, Huang C-C, Xu X, et al. Society of Cardiovascular Anesthesiologists/European Association of Cardiothoracic Anaesthetists Practice Advisory for the Management of Perioperative Atrial Fibrillation in Patients Undergoing Cardiac Surgery. Anesth Analg. 2019;128: 33–42. doi: 10.1213/ANE.0000000000003865 30550473

[20] Weiner ID, Wingo CS. Hypokalemia—consequences, causes, and correction. J Am Soc Nephrol. 1997;8: 1179–1188. Available: http://jasn.asnjournals.org/content/8/7/1179.short. 9219169 10.1681/ASN.V871179

[21] Campbell NG, Allen E, Sanders J, Swinson R, Birch S, Sturgess J, et al. The impact of maintaining serum potassium ≥ 3.6 mEq/L vs ≥ 4.5 mEq/L on the incidence of new-onset atrial fibrillation in the first 120 hours after isolated elective coronary artery bypass grafting—study protocol for a randomised feasibility trial for the proposed Tight K non-inferiority trial. Trials. 2017;18: 1061. doi: 10.1186/s13063-017-2349-x 29282098 PMC5745783

[22] Campbell NG, Allen E, Montgomery H, Aron J, Canter RR, Dodd M, et al. Maintenance of serum potassium levels ≥ 3.6 mEq/L vs ≥ 4.5 mEq/L after isolated elective coronary artery bypass grafting, and the incidence of new-onset atrial fibrillation: pilot and feasibility study results. J Cardiothor Vasc An. 2022; 36: 847–854. doi: 10.1053/j.jvca.2021.06.021 34404592

[23] Amar D, Shi W, Hogue CW, Zhang H, Passman RS, Thomas B, et al. Clinical prediction rule for atrial fibrillation after coronary artery bypass grafting. JACC. 2004;44: 1248–1253. doi: 10.1016/j.jacc.2004.05.078 15364327

[24] Chan A-W, Tetzlaff JM, Gøtzsche PC, Altman DG, Mann H, Berlin JA, et al. SPIRIT 2013 explanation and elaboration: guidance for protocols of clinical trials. BMJ. 2013;346: e7586. doi: 10.1136/bmj.e7586 23303884 PMC3541470

[25] Lip GYH, Nieuwlaat R, Pisters R, Lane DA, Crijns HJGM. Refining clinical risk stratification for predicting stroke and thromboembolism in atrial fibrillation using a novel risk factor-based approach: the euro heart survey on atrial fibrillation. Chest. 2010;137: 263–272. doi: 10.1378/chest.09-1584 19762550

[26] Nashef SAM, Roques F, Sharples LD, Nilsson J, Smith C, Goldstone AR, et al. EuroSCORE II. Eur J Cardiothorac Surg. 2012;41: 734–745. doi: 10.1093/ejcts/ezs043 22378855

[27] Hindricks G, Potpara T, Dagres N, Arbelo E, Bax JJ, Blomström-Lundqvist C, et al. 2020 ESC Guidelines for the diagnosis and management of atrial fibrillation developed in collaboration with the European Association of Cardio-Thoracic Surgery (EACTS)The Task Force for the diagnosis and management of atrial fibrillation of the European Society of Cardiology (ESC) Developed with the special contribution of the European Heart Rhythm Association (EHRA) of the ESC. EHJ. 2020;42: 373–498. ehaa612. doi: 10.1093/eurheartj/ehaa612 32860505

[28] Calkins H, Kuck K-H, Cappato R, Brugada J, Camm AJ, Chen S-A, et al. 2012 HRS/EHRA/ECAS expert consensus statement on catheter and surgical ablation of atrial fibrillation: recommendations for patient selection, procedural techniques, patient management and follow-up, definitions, endpoints, and research trial design: a report of the Heart Rhythm Society (HRS) Task Force on Catheter and Surgical Ablation of Atrial Fibrillation. Developed in partnership with the European Heart Rhythm Association (EHRA), a registered branch of the European Society of Cardiology (ESC) and the European Cardiac Arrhythmia Society (ECAS); and in collaboration with the American College of Cardiology (ACC), American Heart Association (AHA), the Asia Pacific Heart Rhythm Society (APHRS), and the Society of Thoracic Surgeons (STS). Endorsed by the governing bodies of the American College of Cardiology Foundation, the American Heart Association, the European Cardiac Arrhythmia Society, the European Heart Rhythm Association, the Society of Thoracic Surgeons, the Asia Pacific Heart Rhythm Society, and the Heart Rhythm Society. Heart Rhythm. 2012;9: 632–696.e21. doi: 10.1016/j.hrthm.2011.12.016 22386883

[29] Piaggio G, Elbourne DR, Pocock SJ, Evans SJW, Altman DG, Group for the C. Reporting of Noninferiority and Equivalence Randomized Trials: Extension of the CONSORT 2010 Statement. JAMA. 2012;308: 2594–2604. doi: 10.1001/jama.2012.87802 23268518

[30] Campbell NG, Wollborn J, Fields KG, Lip GYH, Ruetzler K, Muehlschlegel JD, et al. Inconsistent Methodology as a Barrier to Meaningful Research Outputs from Studies of Atrial Fibrillation After Cardiac Surgery. J Cardiothor Vasc Anesth. 2022;36: 739–745. doi: 10.1053/j.jvca.2021.10.009 34763979 PMC9901359

