# Correction: Impact of maintaining serum potassium concentration ≥ 3.6mEq/L versus ≥ 4.5mEq/L for 120 hours after isolated coronary artery bypass graft surgery on incidence of new onset atrial fibrillation: Protocol for a randomized non-inferiority trial

**DOI:** 10.1371/journal.pone.0304230

**Published:** 2024-05-20

**Authors:** Niall G. Campbell, Elizabeth Allen, Richard Evans, Zahra Jamal, Charles Opondo, Julie Sanders, Joanna Sturgess, Hugh E. Montgomery, Diana Elbourne, Benjamin O’Brien

There are two instances in the article where the procedure was erroneously referred to as “elective isolated CABG”, when it should have been correctly termed as “isolated CABG”. The first instance is found in the background section of the abstract, and the second is in the initial paragraph of the Trial Population section under Methods. This study also recruited non-elective patients. Therefore, the term ‘elective’ is not applicable and should be disregarded in both instances.
