# Supplementary material for: Impact of maintaining serum potassium concentration ≥ 3.6mEq/L versus ≥ 4.5mEq/L for 120 hours after isolated coronary artery bypass graft surgery on incidence of new onset atrial fibrillation: Protocol for a randomized non-inferiority trial
Source: PLoS One. 2024 Mar 13;19(3):e0296525. doi: 10.1371/journal.pone.0296525 (PMC10936833; doi:10.1371/journal.pone.0296525)
Supplement: S2 File — (DOCX) [file pone.0296525.s002.docx]

**List of Tight K sites:**

St Bartholomew's Hospital, United Kingdom

Wythenshawe Hospital, United Kingdom

King's College Hospital, United Kingdom

Castle Hill Hospital, United Kingdom

Liverpool Heart and Chest Hospital, United Kingdom

Hammersmith Hospital, United Kingdom

Leeds General Infirmary, United Kingdom

Blackpool Victoria Hospital, United Kingdom

St George's Hospital, United Kingdom

Northern General Hospital, United Kingdom

Royal Sussex County Hospital, United Kingdom

John Radcliffe Hospital, United Kingdom

Freeman Hospital, United Kingdom

Basildon University Hospital, United Kingdom

Derriford Hospital, United Kingdom

Aberdeen Royal Infirmary, United Kingdom

Guy’s and St Thomas’, United Kingdom

Nottingham City Hospital, United Kingdom

Bristol Heart Institute, United Kingdom

Royal Infirmary of Edinburgh, United Kingdom

Golden Jubilee Hospital, United Kingdom

Deutsches Herzzentrum der Charité, Berlin, Germany

Westfälische Wilhelms-Universität, Münster, Germany
