# Supplementary material for: Impact of maintaining serum potassium concentration ≥ 3.6mEq/L versus ≥ 4.5mEq/L for 120 hours after isolated coronary artery bypass graft surgery on incidence of new onset atrial fibrillation: Protocol for a randomized non-inferiority trial
Source: PLoS One. 2024 Mar 13;19(3):e0296525. doi: 10.1371/journal.pone.0296525 (PMC10936833; doi:10.1371/journal.pone.0296525)
Supplement: S3 File — (PDF) [file pone.0296525.s003.pdf]

[redacted]

TIGHT K

Prevention of dysrhythmias on the cardiac intensive care unit - does maintenance of high-normal serum potassium levels matter?

Tight K Trial Protocol

Version 3 01/02/2023

Funded by the British Heart Foundation

Sponsored by Barts Health NHS Trust

Managed by London School of Hygiene & Tropical Medicine Clinical Trials Unit

**Full Title**	The TIGHT-K STUDY. Prevention of dysrhythmias on the cardiac intensive care unit - does maintenance of high-normal serum potassium levels matter?
**Short Title/Acronym**	Tight K Trial
**Sponsor**	Barts Health NHS Trust<br>Dr Mays Jawad<br>Director of Research Services<br>Joint Research Management Office<br>5 Walden Street<br>London E1 2EF<br>[redacted]
**REC Reference**	19/LO/1064
**IRAS Reference**	260639
**Chief Investigator**	Prof Ben O'Brien<br>Professor of Perioperative Medicine<br>Consultant in Intensive Care Medicine and Cardiac Anaesthesia<br>Barts Health NHS Trust<br>St Bartholomew's Hospital<br>London<br>EC1A 7DN<br>[redacted]
**Clinical Trials Unit**	Department of Medical Statistics<br>London School of Hygiene & Tropical Medicine<br>Keppel Street<br>London<br>WC1E 7HT<br>[redacted]

Contents

1. GLOSSARY OF TERMS AND ABBREVIATIONS 6

2. SIGNATURE PAGE 7

2.1. Chief Investigator Agreement 7
2.2. Statistician Agreement 7

3. SUMMARY / SYNOPSIS 8

3.1. Protocol Summary 8
3.2. Trial Flowchart 10

4. INTRODUCTION 11

4.1. Background 11
4.2. Feasibility study results 12
4.3. Rationale for the trial 12

5. AIM AND OBJECTIVES 12

5.1. Aim 12
5.2. Hypothesis 12
5.3. Objectives 13
5.3.1. Primary objective 13
5.3.2. Secondary objectives 13

6. TRIAL SETTING AND DESIGN 13

6.1. Setting 13
6.1.1. Site requirements 13
6.1.2. Site and PI responsibilities 13
6.1.3. Site initiation and activation 13
6.2. Trial design 14
6.3. Outcome definitions 14
6.3.1. Definition of AFACS 14
6.3.2. Definition of Holter-Identified AFACS 15
6.3.3. Definition of non-AFACS arrythmias 15
6.4. Primary outcome 15
6.4.1. Adjudication of primary outcome 15
6.5. Secondary outcomes 17

7. SELECTION AND WITHDRAWAL OF PARTICIPANTS 17

7.1. Inclusion criteria 17
7.2. Exclusion criteria 17
7.3. Definition of isolated CABG surgery 17
7.4. Co-enrolment 17
7.5. Withdrawal of participants 18
7.5.1. Criteria for withdrawal from the trial 18
7.5.2. Withdrawing from the trial treatment 18
7.5.3. Withdrawing from trial and/or follow-up 18
7.5.4. Withdrawing from the trial 18
7.5.5. Reporting withdrawal 18

8. TRIAL PROCEDURES 18

8.1. Screening 18
8.1.1. Screening log 19
8.2. Informed consent procedure 19
8.3. Randomisation 19
8.3.1. Intervention arm 20

8.3.2. *Control arm* ... 20
8.3.3. *Blinding* ... 20
8.4. CABG POSTPONEMENT OR CANCELLATION POST-RANDOMISATION ... 20
8.4.1. *CABG rescheduled within 6 months of randomisation* ... 20
8.4.2. *No CABG rescheduled within 6 months of randomisation* ... 20
8.5. TRIAL TREATMENT ... 20
8.5.1. *End of Trial Treatment* ... 21
8.6. HEART RHYTHM MONITORING ... 21
8.6.1. *Holter monitoring after AFACS* ... 21
8.6.2. *Sending Holter monitor data to the core lab* ... 21
8.6.3. *Holter core laboratory* ... 21
8.6.4. *Unused Holter monitors* ... 22
8.7. FOLLOW-UP ... 22
8.8. END OF TRIAL ... 22

9. DATA COLLECTION ... 22

9.1. TRIAL TREATMENT PERIOD ... 22
9.2. TRIAL PROCEDURES TABLE ... 24
9.3. TRIAL PROCEDURES ... 25
9.3.1. *Before surgery* ... 25
9.3.2. *Baseline* ... 25
9.3.3. *Periods 1-5* ... 25
9.3.4. *Discharge (from ICU and hospital)* ... 25
9.3.5. *Clinical follow-up (if carried out)* ... 26
9.3.6. *Follow-up visit 6-months post-surgery* ... 26
9.4. COMPLIANCE AND LOSS TO FOLLOW-UP ... 26
9.4.1. *Loss to follow-up* ... 26
9.4.2. *Compliance* ... 26
9.4.3. *Data Handling and Record Keeping* ... 27

10. SUBSTUDIES ... 27

10.1. BASIC SCIENCE SUBSTUDY ... 27
10.1.1. *Procedure* ... 27
10.1.2. *Sample processing and storage* ... 28
10.2. DATA SCIENCE SUBSTUDY ... 28

11. MONITORING AND AUDITS ... 28

12. SAFETY MONITORING ... 28

12.1. DEFINITION ... 28
12.2. EXPECTED ADVERSE EVENTS ... 29
12.3. UNEXPECTED SERIOUS ADVERSE EVENTS ... 29
12.4. UNEXPECTED NON-SERIOUS ADVERSE EVENTS ... 30
12.5. REPORTING UNEXPECTED ADVERSE EVENTS ... 30
12.5.1. *Assessment of intensity* ... 30
12.5.2. *Assessment of causality* ... 30
12.6. URGENT SAFETY MEASURES ... 31
12.7. ANNUAL SAFETY REPORTING ... 31
12.8. OVERVIEW OF THE SAFETY REPORTING RESPONSIBILITIES ... 31

13. STATISTICAL CONSIDERATIONS ... 31

13.1. POWER CALCULATIONS AND SAMPLE SIZE DETERMINATION ... 31
13.2. TRIAL STATISTICIAN ... 31
13.3. STATISTICAL ANALYSIS ... 32
13.3.1. *Summary of baseline data and flow of participants* ... 32
13.3.2. *Primary and secondary outcome analyses* ... 32
13.3.3. *Exploratory analyses* ... 33

14. ETHICS ... **33**
14.1. Declaration of Helsinki and Good Clinical Practice ... 33
14.2. UK ethics committee review ... 33
14.3. Confidentiality Advisory Group ... 33
15. MANAGEMENT AND OVERSIGHT ... **33**
15.1. Trial Management Group (TMG) ... 33
15.2. Trial Steering Committee (TSC) ... 34
15.3. Data Safety and Monitoring Committee (DSMC) ... 34
15.4. Event Validation Committee (EVC) ... 34
16. FINANCE AND FUNDING ... **34**
17. INDEMNITY ... **34**
17.1. Sponsorship ... 34
17.2. Insurance ... 34
18. DISSEMINATION OF RESEARCH FINDINGS ... **34**
19. REFERENCES ... **35**
APPENDIX 1: AMENDMENT HISTORY ... **37**

1. Glossary of Terms and Abbreviations

AE	adverse event
AF	atrial fibrillation
AFACS	atrial fibrillation after cardiac surgery, incorporating atrial fibrillation, atrial Flutter and atrial tachycardia
AV	atrioventricular
BHF	British Heart Foundation
CABG	coronary artery bypass graft
CAG	Confidentiality Advisory Group
CI	confidence interval
CRF	case report form
CTU	Clinical Trials Unit
DSMC	Data Safety and Monitoring Committee
ECG	electrocardiogram
eCRF	electronic case report form
EQ-5D-5L	EuroQol EQ-5D 5-level questionnaire
Holter monitor	The CAM™ heart rhythm monitor manufactured by Bardy is referred to as a Holter monitor in the protocol
HRA	Health Research Authority
ICH-GCP	International Council for Harmonisation Good Clinical Practice
ICU	Intensive Care Unit
IRAS	Integrated Research Application System
ITT	Intention-to-treat
IV	intravenous
JRMO	Joint Research Management Office
$[K^+]$	potassium concentration
LSHTM	London School of Hygiene & Tropical Medicine
NHS	National Health Service
NSAE	non-serious adverse event
Participant	An individual who takes part in a clinical trial
PI	Principal Investigator
PIS	Participant Information Sheet
RCT	randomised clinical trial
REC	Research Ethics Committee
SAE	serious adverse event
TMG	Trial Management Group
TSC	Trial Steering Committee
WPD	Working Practice Document

2. Signature Page

2.1. Chief Investigator Agreement

The clinical study as detailed within this research protocol **(Version 3, 09/01/2023)**, or any subsequent amendments will be conducted in accordance with the current Research Governance Framework for Health & Social Care (2005), the World Medical Association Declaration of Helsinki (1996) and the current applicable regulatory requirements and any subsequent amendments of the appropriate regulations.

Chief Investigator Name: Prof Ben O'Brien

Chief Investigator Site: Barts Heart Centre

Signature: [redacted] **Date:** 01/02/2023

2.2. Statistician Agreement

The clinical study as detailed within this research protocol **(Version 3, 25/05/2022)** or any subsequent amendments will be conducted in accordance with the current Research Governance Framework for Health & Social Care, the World Medical Association Declaration of Helsinki (1996), Principles of ICH E6-GCP, ICH E9 - Statistical principles for Clinical Trials, ICH E10 - Choice of Control Groups and the current applicable regulatory requirements and any subsequent amendments of the appropriate regulations.

Statistician Name: Professor Elizabeth Allen

Statistician Site: London School of Hygiene and Tropical Medicine

Signature: [redacted] **Date:** 01/02/2023

3. Summary / Synopsis

3.1. Protocol Summary

Short Title	Tight K Trial
Design	Multicentre, non-inferiority randomised clinical trial
Sites	15-25 NHS Hospitals<br>1-5 sites in Germany (German sites are self-sponsored)
Aim	To determine whether a strategy of maintaining serum potassium levels at ≥3.6 mEq/L is non-inferior to a strategy of usual treatment (≥4.5 mEq/L) on the occurrence of new onset atrial fibrillation after cardiac surgery (AFACS) in patients undergoing isolated coronary artery bypass graft (CABG) surgery
Primary outcome	The presence of new onset AFACS that is both clinically detected and electrocardiographically confirmed (on either electrocardiogram [ECG], telemetry or Holter monitoring) until hour 120 after initial admission to ICU/post-operative care facility, discharge from hospital, or with occurrence of a site-reported episode of AFACS (whichever occurs first).
Secondary outcomes	• The incidence of new onset AFACS detected on Holter monitor until hour 120 after initial admission to ICU/post-operative care facility or discharge from hospital, whichever occurs first.<br>• The incidence of at least one episode of AFACS (clinically identified) or Holter-identified AFACS (where none identified clinically) until hour 120 after initial admission to ICU/post-operative care facility or discharge from hospital, whichever occurs first.<br>• Number of patients experiencing at least one episode of a non-AF arrhythmia, identified on Holter monitors until hour 120 after initial admission to ICU/post-operative care facility, or discharge from hospital, whichever occurs first.<br>• In-patient mortality<br>• 6-month mortality<br>• Critical care length of stay<br>• Hospital length of stay<br>• Costs relating to potassium therapy<br>• Quality of life at 6 months
Inclusion criteria	1. Scheduled to have isolated CABG surgery<br>2. Patient in sinus rhythm
Exclusion criteria	1. Age less than 18 years<br>2. Previous history of Atrial Fibrillation, Atrial Flutter and/or Atrial Tachyarrhythmia<br>3. Pre-operative high-degree atrioventricular (AV) block *(defined as* Mobitz type 2 second degree AV block *or* complete heart block*)*

	4. Pre-operative serum [K^+] greater than 5.5 mEq/L<br>5. Current or previous use of medication for the purposes of cardiac rhythm management<br>6. Dialysis-dependent end-stage renal failure<br>7. Concurrent patient involvement in another clinical study assessing cardiac rhythm post-operative interventions<br>8. Unable to provide informed consent
Number of Participants	1684 participants, approx. 842 in each trial arm
Trial arms	**Intervention:** Serum potassium levels maintained at ≥3.6 mEq/L ('Relaxed'). Those randomised to the 'Relaxed' Group will receive [K^+] supplementation when their serum [K^+] drops below 3.6mEq/L<br>**Control:** Serum potassium levels maintained at ≥4.5 mEq/L ('Tight'). Patients randomised to the 'Tight' group will receive [K^+] supplementation when their serum [K^+] falls below 4.5 mEq/L.
Trial duration	Trial duration is expected to be 5 years and 3 months (63 months).<br>Recruitment is projected to continue until Q4 December 2023.

3.2. Trial Flowchart

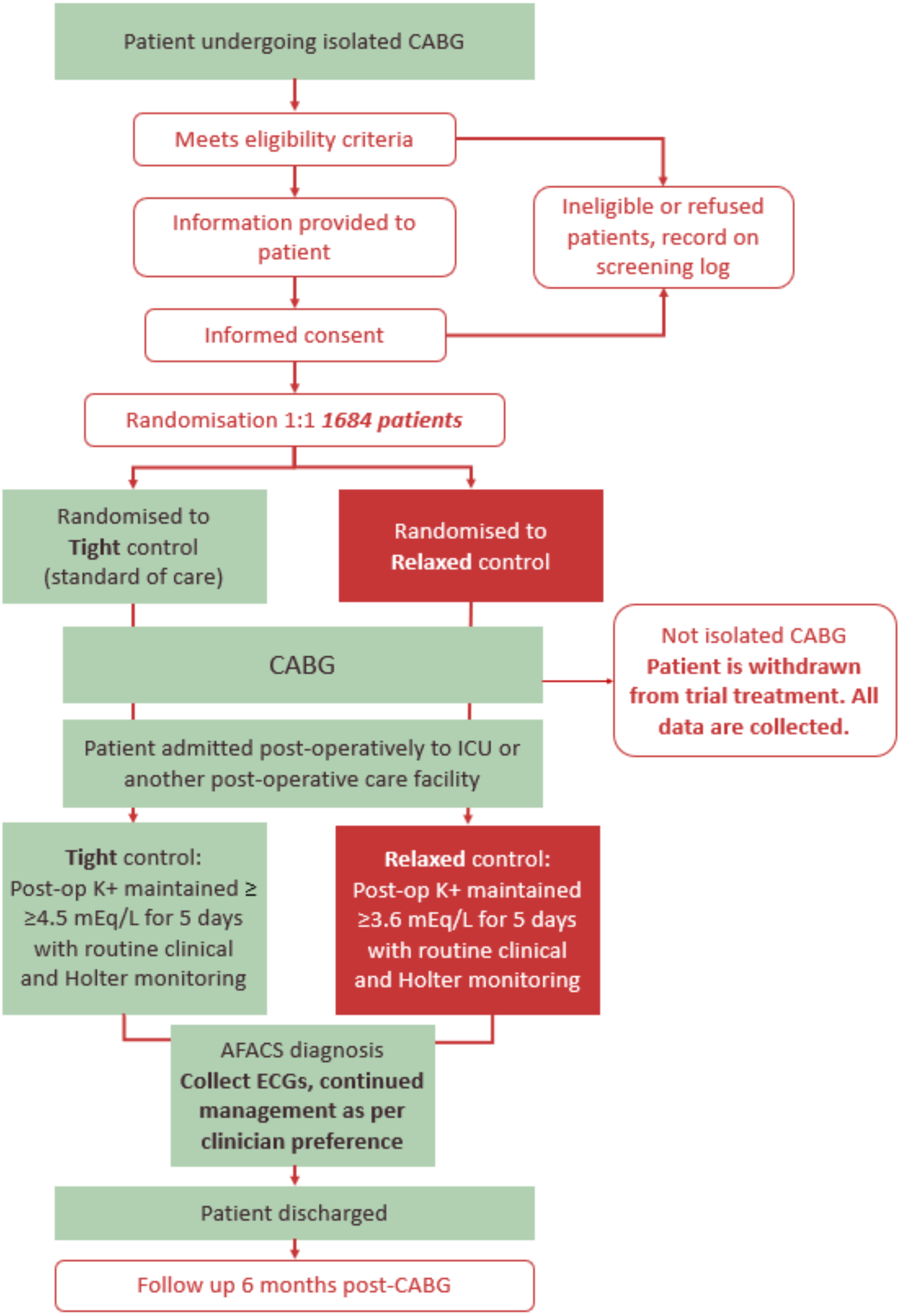

4. Introduction

4.1. Background

At least one in three patients is affected by atrial fibrillation after cardiac surgery (AFACS), with most episodes occurring in the first five post-operative days[1-3]. AF occurrence is associated with increased morbidity, short and long-term mortality[3-6], intensive care unit (ICU) and hospital stay[7, 8], and cost of care[9]. Persistence of these associations, after adjustment for potential confounding factors, suggests that they may be causal[10]. The incidence and prevalence of AFACS and its associated costs are expected to increase as the surgical population ages[11]. Extensive effort is undertaken to prevent AF after cardiac surgery from occurring, but clinical practice in this area is highly variable and the evidence base for most interventions is sparse[12, 13].

Potassium plays an important role in cardiac electrophysiology[14]. Serum potassium concentrations ([K^+]) are commonly low following cardiac surgery[15], and appear marginally lower in those suffering atrial arrhythmias in non-surgical cohorts[16]. Despite an absence of proof that this association is causal, efforts to maintain serum [K^+] in the 'high-normal' range (≥4.5 mEq/L), as opposed to just intervening if potassium drops below its lower 'normal' threshold (<3.6 mEq/L), are considered 'routine practice' for AF prevention in post-surgical patients in many centres across the world[17]. From the (unpublished) data from our British Heart Foundation (BHF) funded Tight-K Feasibility Study, all 160 patients would have required at least one dose of potassium to supplement their levels to this high-normal range and 45.5% of all serum [K^+] measurements were below 4.5mEq/L at some point. Data from the same pilot study show a median number of potassium doses given in the 'tight' group (high-normal serum potassium target) of seven, compared to a median of one, with most patients not receiving any potassium supplementation at all, in the 'relaxed' group. We did, for the first time ever, show that the practice does achieve a separation in serum potassium levels between the two groups, so the protocol is indeed effective in achieving higher serum potassium levels.

The efficacy of the practice of maintaining high-normal serum potassium levels for the prevention of AFACS, however, remains unproven and data supporting it are extremely limited, being derived from observational studies rather than randomised trials[17]. Indeed, no data exist to demonstrate that maintaining a high-normal potassium level is beneficial in these circumstances, or that aggressive replenishment of potassium in these patients improves outcome[18].

Meanwhile, potassium supplementation may cause discomfort or harm. Routine central venous potassium administration in the early post-operative period, when oral supplementation is not possible, is time-consuming, costly and associated with clinical risk: rapid infusion can prove fatal[19], and leaving central venous catheters in situ for the sole purpose of potassium replacement increases infection risk[20]. Oral replacement (when feasible) is commonly associated with profound nausea and gastrointestinal side effects, and is very poorly tolerated by patients[21, 22]. The annual costs of intravenous potassium exceed those for other drugs in many cardiac surgical units due to the large quantities administered[23]. Nursing time (e.g. for drug checks and administration) will add to this cost.

4.2. Feasibility study results

We recruited 160 patients between 28 August 2017 and 24 April 2018. The average recruitment rate was thus 20 patients per month over two sites. Of 601 screened patients, 24% were recruited, 48% were eligible but not recruited and 27% were ineligible. Randomisation was acceptable and was successful in all recruited patients. Provisional data on the need for potassium administration showed that in the relaxed arm, 19 out of 79 patients (24.1%) had at least one measurement below 3.6mEq/L and therefore required potassium supplementation. In the tight arm, 80 out of 81 patients (98.8%) with potassium data had at least one measurement below 4.5mEq/L and required potassium. Data available on potassium protocol violations across both the Tight and Relaxed Groups demonstrated a rate of 9.8% (283/2886), defined as a serum potassium measurement that either resulted in (1) supplementation being administered when it should have been withheld or (2) supplementation being withheld when it should have been administered.

The follow-up rate was 91.3% at 28 days post-surgery. Twelve patients (7.5%) were not followed up post-discharge and 2 patients (1.3%) died prior to 28 days post-surgery.

4.3. Rationale for the trial

The routine maintenance of serum [K^+] ≥4.5 mEq/L is of unproven efficacy, may be unpleasant or hazardous for patients, and is costly. We shall address this issue, performing the first appropriately powered non-inferiority multicentre randomised trial of a strategy of 'relaxed' potassium supplementation versus a strategy of 'tight' potassium supplementation, which is defined as standard of care. The findings will have important consequences for patients and clinicians, regardless of whether or not potassium supplementation is found to be non-inferior for the prevention of AF after cardiac surgery. A survey of practice patterns in Europe and North America suggests that there is genuine equipoise, with 67% of caregivers practising in Europe reporting that their institution has a protocol for maintaining high-normal serum potassium levels after cardiac surgery[12, 13]. So one in three do not.

The Tight K Trial will set out to test the hypothesis that AFACS will be no more common after coronary artery bypass graft (CABG) surgery when serum potassium levels are maintained ≥3.6 mEq/L as when they are maintained ≥4.5 mEq/L.

5. Aim and Objectives

5.1. Aim

The aim of the Tight K Trial is to determine whether a strategy of maintaining serum potassium levels at ≥3.6 mEq/L is non-inferior to a strategy maintaining levels ≥4.5 mEq/L on the occurrence of new onset AFACS post-surgery in patients undergoing CABG surgery.

5.2. Hypothesis

New onset AFACS will be no more common after CABG surgery as when serum potassium levels are maintained ≥3.6 mEq/L than when they are maintained ≥4.5 mEq/L.

5.3. Objectives

5.3.1. Primary objective

- Assess whether new onset AFACS is as prevalent in the first 120 hours after isolated CABG surgery, when a strategy of serum [K^+] maintenance of ≥4.5 mEq/L, as with a strategy of serum [K^+] maintenance of ≥3.6 mEq/L.

5.3.2. Secondary objectives

- Estimate the AFACS burden on recovering CABG surgery patients
- Estimate the cost of delivering potassium interventions

6. Trial setting and design

6.1. Setting

6.1.1. Site requirements

- Perform CABG surgeries on site
- Compliance with all responsibilities as stated in the Tight K Model Agreement for Non-Commercial Research
- Compliance with all requirements of the trial protocol, including the trial treatment and follow-up schedules
- Compliance with the Research Governance Framework for Health and Social Care and International Council for Harmonisation Guidelines on Good Clinical Practice (ICH-GCP)

6.1.2. Site and PI responsibilities

- Identify at least one local Principal Investigator (PI)
- Ensure agreement is obtained to incorporate the Tight K Trial into routine post-surgical and critical care clinical practice across multiple disciplines and relevant post-operative ward environments
- Adherence with the most recent approved version of the trial protocol
- Ensure training of relevant site staff in accordance with the trial protocol and ICH-GCP requirements
- Establish workflows to randomise a high proportion of eligible patients and maintain a screening log
- Agree to adhere to individual patient randomisation allocations
- Agree to timely data collection, entry and validation
- Agree to prompt notification of all adverse events

6.1.3. Site initiation and activation

The following must be in place before a site can be activated for recruitment:

- Completed site initiation visit
- All relevant institutional approvals (e.g. local confirmation of capacity and capability)
- Fully signed Tight K Model Agreement for Non-Commercial Research
- Completed Delegation Log and Training Logs

Once the London School of Hygiene & Tropical Medicine (LSHTM) Clinical Trials Unit (CTU) have confirmed that all necessary documentation is in place, a site activation email will be issued to the PI, at which point, the site may start to screen for eligible patients.

All local staff (i.e. PIs, local investigators, research teams) involved in the conduct of the trial must be listed and signed off on the Delegation Log, once trained to carry out their delegated duties. The Delegation Log should be copied and sent to the Tight K Trial Team at the LSHTM CTU whenever changes are made

6.2. Trial design

The Tight K Trial is a pragmatic, multicentre, non-inferiority randomised clinical trial (RCT).

6.3. Outcome definitions

6.3.1. Definition of AFACS

AFACS will be defined as an episode of atrial fibrillation, flutter or tachyarrhythmia. The minimum duration of atrial fibrillation, flutter or tachyarrhythmia on an ECG trace and/or rhythm strip/telemetry required to establish the diagnosis of AFACS is at least 30 seconds, or an entire 12-lead ECG.

Local guidelines will be used to diagnose any of the three contributing atrial dysrhythmias.

ECG criteria for ***Atrial fibrillation*** are[24]:

1. Absolutely irregular RR intervals in the absence of complete AV block
2. No distinct P waves on the surface ECG
3. An atrial cycle length (when visible) that is usually variable and less than 200ms.

Atrial flutter refers classically to a pattern of regular tachycardia with atrial rate ≥ 240 beats per minute lacking an isoelectric baseline between deflections. A characteristic ECG 'sawtooth' pattern may be present in leads II, III and/or aVF, but is not seen in all. Continuous undulation of the atrial complex without a sawtooth appearance can sometimes be identified.

Atrial tachycardias are regular atrial rhythms with a sudden onset / offset at a constant atrial rate ≥ 100 beats per minute, with an isoelectric baseline between deflections. The P-wave morphology is different to that of sinus rhythm and the ventricular rate is usually regular.

It can be challenging to discriminate between atrial flutter and atrial tachyarrhythmias on electrocardiographic grounds alone. For the purposes of this study, they will thus be categorised together.

6.3.1.1. Potassium restrictions on patients with AFACS

Once a participant has a site-reported period of AFACS, there will be no restriction on potassium supplementation and the participant should be treated according to current practice. A copy of the ECG, and/or rhythm strip/telemetry printout should be stored locally for verification.

6.3.2. Definition of Holter-Identified AFACS

Holter-Identified AFACS will be defined as an episode of atrial fibrillation, flutter or tachyarrhythmia (as defined above) lasting ≥ 30 seconds that is detected on Holter monitoring[24] but not reported by site as AFACS. Episodes lasting <30 seconds will not be counted for the purposes of the secondary endpoints.

6.3.3. Definition of non-AFACS arrythmias

Non-AF arrhythmias are defined as;

1. Non-AF supraventricular arrhythmia of ≥ 30 seconds
2. Ventricular tachycardia/fibrillation (defined as more than 3 beats at ≥ 100bpm)
3. Mobitz type 2 block (any duration)
4. Complete heart block (any duration)
5. Other ventricular pause ≥ 3 seconds

6.4. Primary outcome

The presence of new onset AFACS that is both clinically detected and electrocardiographically confirmed (on either electrocardiogram [ECG], telemetry or Holter monitoring) until hour 120 after initial admission to ICU/post-operative care facility, discharge from hospital, or with occurrence of a site-reported episode of AFACS (whichever occurs first).

6.4.1. Adjudication of primary outcome

Episodes of clinically detected AFACS will be reviewed by an independent event validation committee (EVC) who are blinded to treatment allocation. The EVC will adjudicate events according to a hierarchy of evidence. The premise of the hierarchy approach is that site-reported AFACS is adjudicated against the best-available evidence.

6.4.1.1. Primary outcome adjudication outcomes

Category I Evidence: ECG/telemetry/rhythm strip printout provided by the site

- If the ECG/rhythm strip is readable and confirms an uninterrupted episode of atrial fibrillation, atrial flutter or atrial tachyarrhythmia the event will be classified as "**Confirmed AFACS**"
- If the ECG/rhythm strip is readable but there is no evidence of an uninterrupted episode of atrial fibrillation, atrial flutter or atrial tachyarrhythmia then proceed to reviewing Category II evidence.
- If the ECG/rhythm strip is not available, not readable or evidence of an uninterrupted episode of atrial fibrillation, atrial flutter or atrial tachyarrhythmia is inconclusive then proceed to reviewing Category II evidence.

Category II evidence: ECG data collected by the Holter monitor

- If an episode of atrial fibrillation, atrial flutter or atrial tachyarrhythmia lasting ≥ 30 seconds is recorded on the Holter monitor the event will be classified as "**Confirmed AFACS**".

- If Holter data is not available, there is insufficient data due to gaps in the recording, or the data is of insufficient quality, then proceed to reviewing Category III evidence
- If Holter data is of adequate quality and covers the full period of 120 hours (or up to discharge if sooner) and an uninterrupted episode of atrial fibrillation, atrial flutter or atrial tachyarrhythmia lasting ≥ 30 seconds is not recorded on the Holter monitor the event will be classified as “**No AFACS**”.

Category III evidence: Supporting evidence in hospital notes and data collected in the electronic case report form (eCRF)

- The committee will review circumstantial supporting evidence from the eCRF, such as administration of amiodarone or DC/chemical cardioversion, alongside patient notes. The committee will decide based on the level of evidence available whether the event can be classified as either “**Confirmed AFACS**” or “**No AFACS**”.
- If no hospital notes are available or there is insufficient evidence to confirm or refute the presence of AFACS, the event will be classified as “**Probable AFACS**”.

Episodes adjudicated as either Confirmed AFACS or Probable AFACS will be included in the primary endpoint. Episodes adjudicated as No AFACS will not be included in the primary endpoint.

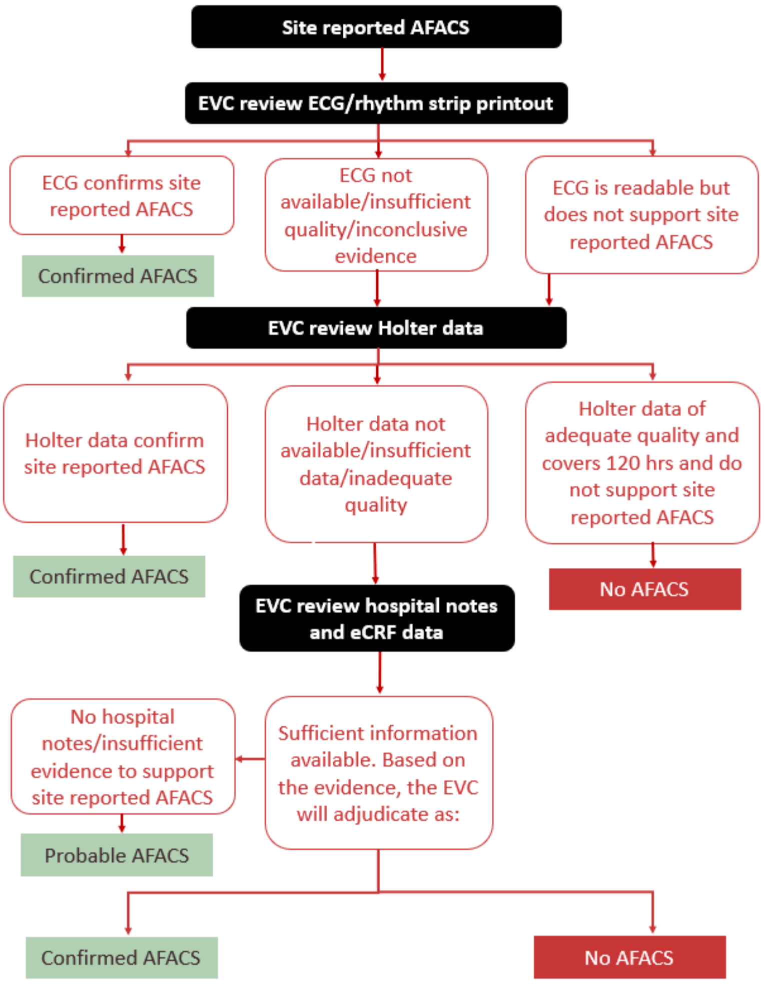

6.5. Secondary outcomes

- The incidence of new onset AFACS detected on Holter monitor until hour 120 after initial admission to ICU/post-operative care facility or discharge from hospital, whichever occurs first.
- The incidence of at least one episode of AFACS (clinically identified) or Holter-identified AFACS (where none identified clinically) until hour 120 after initial admission to ICU/post-operative care facility or discharge from hospital, whichever occurs first.
- The number of patients experiencing at least one episode of a non-AF arrhythmia, identified on Holter monitors until hour 120 after initial admission to ICU/post-operative care facility, discharge from hospital – whichever occurs first.
- In-patient mortality
- 6-month mortality
- Critical care length of stay
- Hospital length of stay
- Costs relating to purchasing and administering potassium therapy.
- Quality of life at 6 months

7. Selection and withdrawal of participants

7.1. Inclusion criteria

1. Scheduled to have isolated CABG surgery (see section 7.3 for definition of isolated CABG)
2. Patient in sinus rhythm

7.2. Exclusion criteria

1. Age less than 18 years
2. Previous history of Atrial Fibrillation, Atrial Flutter and/or Atrial Tachyarrhythmia
3. Pre-operative high-degree atrioventricular (AV) block *(defined as* Mobitz type 2 second degree AV block *or* complete heart block*)*
4. Pre-operative serum [K^+] greater than 5.5 mEq/L
5. Current or previous use of medication for the purposes of cardiac rhythm management
6. Dialysis-dependent end-stage renal failure
7. Concurrent patient involvement in another clinical study assessing cardiac rhythm post-operative interventions
8. Unable to provide informed consent

7.3. Definition of isolated CABG surgery

Isolated CABG surgery is defined as CABG surgery without any additional cardiac or vascular procedure during the same operation. The harvesting of veins or arteries (e.g. internal mammary artery or radial artery) for the purposes of grafting is part of an isolated CABG procedure and is not a barrier to inclusion.

7.4. Co-enrolment

Co-enrolment with observational studies is permitted in Tight K.

Co-enrolment is prohibited in trials assessing cardiac rhythm post-operative interventions.

Co-enrolment with other interventional trials will be assessed by the Trial Management Group (TMG) on a case-by-case basis, with input from the Trial Steering Committee (TSC) where necessary.

7.5. Withdrawal of participants

7.5.1. Criteria for withdrawal from the trial

A participant may decide to withdraw from the trial at any time without prejudice to their future care.

7.5.2. Withdrawing from the trial treatment

Participants who withdraw from the trial treatment will be treated according to standard clinical care. They will remain in the trial and will continue to be followed up by the research team unless otherwise indicated by the participant.

7.5.3. Withdrawing from trial and/or follow-up

Participants may decide to withdraw from any further contact by the research team. In this case data will be collected remotely, unless otherwise indicated by the participant.

7.5.4. Withdrawing from the trial

Participants who withdraw from the trial while in hospital will be treated according to standard clinical care. Participants who withdraw from the trial after discharge from hospital will be followed up as per standard clinical care by local clinical team. Participants will be encouraged to allow data that have been collected before withdrawal to be used in the analyses. However, if consent to use already collected data is also withdrawn, then these data will be discarded. There will be no further follow-up from the research team**.**

7.5.5. Reporting withdrawal

The LSHTM CTU should be informed by email if a participant has withdrawn from the trial, from trial treatment or from follow-up. Once informed the Tight K CTU will complete a withdrawal form on the trial electronic case report form (eCRF).

8. Trial procedures

8.1. Screening

Staff at the participating sites will identify patients who are scheduled to have a CABG procedure from hospital waiting lists. If patients are scheduled to have an isolated CABG procedure, then their notes will be reviewed to confirm that they are eligible to participate. Research staff will approach patients at their scheduled pre-assessment appointment or prior to their scheduled hospital appointment via post, telephone or email to discuss the study.

8.1.1. Screening log

Sites will complete a screening log for all patients screened for the trial. This will include patients who are randomised, who met one or more of the exclusion criteria and who were eligible but not randomised.

Anonymised screening information will be sent monthly to the LSHTM CTU, a template screening outcome log will be provided to each site.

8.2. Informed consent procedure

Patients will be given a copy of the patient information sheet (PIS). The PI or another delegated member of the direct care team, or research team if that is not practicable, will approach the patient and the patient will be given the opportunity to discuss the trial and have any queries answered. Written consent to participation in the trial will be obtained on a consent form. A baseline health questionnaire, the EuroQol EQ-5D 5-level questionnaire (EQ-5D-5L), will be completed by the patient at this time.

One environment where recruitment may commonly occur will be the pre-operative hospital appointment prior to the planned surgery date. At this appointment, the PI or another delegated member of the research team will discuss the study further and answer any questions the patient may have. The research team should ensure that that the PIs or another delegated member of the direct care team, or research team, is available to the patient prior to the pre-operative assessment if consent / recruitment is going to occur at that meeting.

Patients attending for surgery who have been transferred from another hospital may not have a pre-assessment appointment before they arrive at the hospital. They should be given a copy of the PIS to read as soon after arrival as possible before discussing the study in greater detail with the research team.

It is recommended that patients are allowed 24 hours to consider whether or not to take part in the study.

8.3. Randomisation

Patients who have consented to take part will be allocated using an online randomisation system to receive either ‘tight’ or ‘relaxed’ potassium control.

The participant must be randomised prior to admission to the ICU, or other post-operative care facility.

Randomisation may occur prior to the CABG procedure, but it is recommended to randomise as late as practicable.

Treatment allocation will be random and in a 1:1 ratio between the two groups. The randomisation allocation sequence will be computer generated using randomly permuted blocks of varying size and stratified by participating site.

8.3.1. Intervention arm

Those randomised to the 'Relaxed' Group will receive [K^+] supplementation only if their serum [K^+] drops below 3.6mEq/L.

8.3.2. Control arm

Patients randomised to the 'Tight' group will receive [K^+] supplementation when their serum [K^+] falls below 4.5 mEq/L (current practice).

8.3.3. Blinding

Blinding patients and clinical staff to the treatment allocation is not possible. The analysis of Holter monitor data will be carried out blind to the treatment allocation.

8.4. CABG postponement or cancellation post-randomisation

8.4.1. CABG rescheduled within 6 months of randomisation

If the CABG is likely to be rescheduled the participant will remain in the trial, awaiting the new date. The randomised allocation received on the original date of surgery should be carried out on the new date of CABG, the allocation can be verified using either the randomisation system or by checking with the Tight K CTU. Participants must not be randomised again.

Consent must not be assumed to carry over after any delay to surgery, the participant should be asked if they give ongoing informed consent to continue in the trial. A new consent form does not need to be completed.

If the delay exceeds 4 weeks, the baseline data should be reconfirmed or updated at the point of the new surgery date.

Follow-up should occur 6 months after the new date of surgery.

8.4.2. No CABG rescheduled within 6 months of randomisation

If CABG has not been rescheduled by 6 months from the date of randomisation the patient should be followed up at that point.

If CABG is rescheduled after the 6 month follow-up has taken place the patient should be re-screened for eligibility, and if found to be eligible for the trial may be re-consented and re-randomised as a new patient.

8.5. Trial treatment

The trial treatment will start when participants are admitted to the ICU or another post-operative care facility after their surgery. Trial treatment should only commence if the participant is in sinus or paced rhythm on arrival at the ICU.

The participant will undergo regular blood investigations, as per current practice. The frequency of [K^+] monitoring will be according to local protocols, clinician/nursing staff preference and clinical need. All other treatments will be given according to standard clinical care and clinician’s preference.

The administration route used for all potassium replacement will be prescribed according to clinician preference and given according to existing standardised protocols. This may include intravenous (IV) or oral potassium formulation, administration of potassium-rich nasogastric feeding regimens, recommending the consumption of potassium-rich foods or avoidance of potassium losing drugs.

The use of IV magnesium, beta-blockers and anti-dysrhythmic agents will be as per current practice in both groups.

If the participant had surgery but did not undergo an isolated CABG, the patient should be withdrawn from trial treatment and treated according to standard of care. The patient is followed up as normal with all data collected for the full trial treatment period including Holter monitor data and completion of the 6-month follow-up visit.

8.5.1. End of Trial Treatment

The trial treatment period will end 120 hours (5 days) after initial admission to ICU/post-operative care facility, discharge from hospital, or with occurrence of a site-reported episode of AFACS – whichever occurs first. Participants should be treated according to local potassium protocols as soon as the trial treatment period ends.

8.6. Heart Rhythm monitoring

In addition to the usual care, participants will also be asked to wear an external heart rhythm monitor for up to 120 hours (5 days) following their CABG surgery. This will monitor their heart rhythm for any irregular heart rhythms conditions, such as AF. Participants will wear the Carnation Ambulatory Monitor (CAM™) manufactured by Bardy. For simplicity, in the protocol and patient documents we refer to this heart rhythm monitor as a Holter monitor. The Holter monitor will be applied just before the patient arrives on the intensive care unit, or as soon as possible after their arrival. Time and date of monitor application to the patient must be documented in the diary card included in the monitor packaging.

8.6.1. Holter monitoring after AFACS

In the event of an episode of site-reported AFACS, Holter monitoring will continue until the end of the 120 hours or discharge from hospital. However, if the Holter monitor is removed after a site-reported period of AFACS, e.g. for cardioversion, it does not need to be replaced.

8.6.2. Sending Holter monitor data to the core lab

Holter monitors and the diary card will be posted, or their data uploaded, to the core laboratory group based at Wythenshawe Hospital, Manchester University NHS Foundation Trust. The monitor and diary card should be returned to the core lab as soon as possible after the end of the 120 hours intervention period.

8.6.3. Holter core laboratory

Holter monitor data will be reviewed by the core laboratory. Pseudonymised patient data will be analysed blinded to participant allocation, after the acute episode of care and will only be reviewed post-hoc.

If the core lab analysis flags up any clinically significant dysrhythmia, then these will be fed back via the CTU to the participant's site PI and copied to the site research team, as and when that information becomes available. It is important to note that in clinical practice, Holter monitor data are diagnostic, but not prognostic, and the way they are used here in the context of a research trial is no different.

8.6.4. Unused Holter monitors

The Holter monitors remain trial property. Any unused Holter monitors must be returned to the core lab once recruitment activity has ended at each site.

8.7. Follow-up

Site data collection will continue until hour 120 after initial admission to ICU/post-operative care facility or discharge from hospital. Thereafter all participants will be followed up 6 months (+/- 1 month) after surgery. If surgery did not proceed, minimal data will be collected up to discharge and the participant should be followed up at six months post-randomisation.

The six months follow-up will occur either in person, via a telephone call, email or post. An EQ-5D-5L questionnaire will be completed by the patient at this time. Participants will be asked to provide information about further episodes of AFACS and other heart rhythm problems, and stroke after their hospital discharge, if known.

Participants will also be followed up remotely via NHS England and other central UK NHS bodies for any hospitalisations for AFACS between discharge and 6 months post-surgery.

8.8. End of trial

The end of the trial is defined as last participant, last follow-up.

9. Data collection

Data collected for all participants will include collation of adverse events attributed to [K^+] replacement, including gastrointestinal symptoms from oral [K^+] replacement. Medication at hospital discharge will be collated, including whether anticoagulation is commenced for AFACS. Additional staff time for delivering the intervention will be recorded on site visits and will be informed by expert clinical view.

Detailed information will be collected on the resource use associated with delivering each protocol, including the total number of replacement [K^+] interventions and the number of tests for monitoring potassium levels.

9.1. Trial treatment period

The trial treatment period commences when the participant is admitted to ICU or another post-operative care facility after their surgery. The participant's inclusion into the trial and randomised allocation must be clear upon admission to ICU or another post-operative care facility.

Period 1 (0–24 hours post-admission to ICU)
Period 2 (24-48 hours post-admission to ICU)
Period 3 (48-72 hours post-admission to ICU)
Period 4 (72-96 hours post-admission to ICU)
Period 5 (96-120 hours post-admission to ICU)
Clinical Follow-up (6 weeks (+/- 1 month) post-CABG surgery) *Basic Science sub-study only*
Follow-up (6 months (+/- 1 month) post-CABG surgery)

9.2. Trial Procedures Table

	Before Surgery	Day of Surgery	ICU Stay (commences on admission to ICU)					Discharge	Follow-up clinic appointment*	Follow-up visit 6-months post-surgery
			Period 1	Period 2	Period 3	Period 4	Period 5			
Review of eligibility criteria	X									
Consent	X									
Baseline	X									
Randomisation		X								
Trial treatment			X	X	X	X	X			
Holter Monitoring			X	X	X	X	X			
Clinical events			X	X	X	X	X	X		X
Safety monitoring			X	X	X	X	X	X		X
EQ-5D-5L	X									X
Blood sample *(Basic Science substudy only)*	X		X		X		X		X	

*** If applicable, this is not an additional research visit**

9.3. Trial procedures

9.3.1. Before surgery

- Consent
- Quality of life questionnaire (EQ-5D-5L)
- *Basic science substudy only: 10ml blood sample.*

9.3.2. Baseline

- Age
- Gender
- Ethnic origin
- Cardiac medication and indication (including beta-blockers, calcium channel blockers, ACE-inhibitors, Angiotensin II reception blocker, aldosterone antagonists, anticoagulation)
- Medical history: family history of dysrhythmia, chronic obstructive pulmonary disease/lung disease, diabetes mellitus (and type), hypertension, myocardial infarction, chronic kidney disease, transient ischaemic attack or stroke / cerebrovascular accident
- Imaging data: Left ventricular ejection fraction, left atrial size and mitral regurgitation or stenosis (defined as moderate or worse)
- CHADSVASC score will be calculated

9.3.3. Periods 1-5

- Potassium blood readings (measured as per clinician preference and local pathways)
- Potassium administration
- Clinically significant pacing modes
- Resternotomy
- Duration of central venous lines left in situ
- Clinical events, including:
    - AFACS and date/time of event
        - ECG and/or rhythm strip/telemetry printout stored in source documentation (in the event of AFACS)
    - Non-AF supraventricular tachycardia (equal or greater than 30 seconds)
    - Ventricular tachycardia/fibrillation (more than 3 beats at >100 bpm)
    - Mobitz type 2 block
    - Complete heart block
    - Other ventricular pause of greater than or equal to 3 seconds if not described above
- Safety monitoring
- *Basic science substudy only: 10ml blood samples during periods 1, 3 and 5.*

9.3.4. Discharge (from ICU and hospital)

- ICU length of stay

- Hospital length of stay
- Medications
- Clinical events, including:
    - AFACS (yes/no)
    - Non-AF supraventricular tachycardia (equal or greater than 30 seconds)
    - Ventricular tachycardia/fibrillation (more than 3 beats at >100 bpm)
    - Mobitz type 2 block
    - Complete heart block
    - Other ventricular pause of greater than or equal to 3 seconds if not described above
- Safety monitoring

9.3.5. Clinical follow-up (if carried out)

- *Basic science substudy only: 10ml blood sample.*

9.3.6. Follow-up visit 6-months post-surgery

- Quality of life questionnaire (EQ-5D-5L)
- Clinical events, including:
    - AFACS and date/time of event
    - Non-AF supraventricular tachycardia (equal or greater than 30 seconds)
    - Ventricular tachycardia/fibrillation (more than 3 beats at >100 bpm)
    - Mobitz type 2 block
    - Complete heart block
    - Other ventricular pause of greater than or equal to 3 seconds if not described above
- Safety monitoring

9.4. Compliance and loss to follow-up

9.4.1. Loss to follow-up

The majority of participants remain in hospital for 5 days after their CABG surgery, so loss to follow up over that period is unlikely.

The participants will subsequently be followed up at 6 months following CABG surgery. Participants will be given an ID card to remind them they are taking part in the study and to optimise event reporting during follow-up to the trial team.

9.4.2. Compliance

Given that a large number of different health providers care for post-operative patients in different hospital locations, it is conceivable that protocol violations will occur. These violations may either result from a patient from the 'Relaxed' group being treated as if they are in the 'Tight' Group, or vice versa.

Patients, as they are not blinded, can play a role in reminding staff which to group they have been allocated. Patients will wear colour coded wristbands to help staff to identify which group they have been allocated to. The trial is not blinded to caregivers on the ICU/surgical

ward so there is always a risk of concomitant treatment bias, but training will be put in place to minimise this.

It is critical for the success of the trial that junior doctors and nursing staff in all the post-operative areas where patients may spend time are informed about the trial's goals and protocols. It is more likely that protocol violations will occur once the patient leaves the ICU and arrives on the post-operative step down ward. These wards have lower staff-to-patient ratios and a higher turnover of staff members. Data will be reviewed for each site's first 5 patients and then every 3 months on a rolling basis. If there is an issue with one of the patients from the sites first 5 recruited, that site will be monitored more closely, and the next 5 patients recruited will also be reviewed.

9.4.3. Data Handling and Record Keeping

Data will be entered onto an online database and stored securely on Rackspace servers; http://www.rackspace.co.uk and managed by Sealed Envelope™. Data will be kept for 20 years following completion of the trial.

Pseudonymised patient data will be stored on non-networked PCs at the Core Lab at Wythenshawe Hospital, Manchester University NHS Foundation Trust, with regular external code-locked USBs (with or without secure cloud back-up).

The data controller for the trial is the Chief Investigator (Barts Health NHS Trust are the data controller's organisation) and the data processors are LSHTM and Wythenshawe Hospital.

Patient data will be kept confidential and managed in accordance with the Data Protection Act (2018), NHS Caldecott principles, the Research Governance Framework for Health and Social Care, and the conditions of Research Ethics Committee Approval.

10. Substudies

10.1. Basic Science substudy

The Tight K Basic Science substudy will assess the role of inflammatory markers and genetic components detectable in blood on the development of AFACs. This will improve understanding of the processes which lead to AFACS at a cellular level and inform preventative measures.

Participation in the Tight K Basic Science substudy is only available to patients scheduled to have their CABG procedure at Barts Health NHS Trust.

Patients may still enrol in the main trial without enrolling in the Basic Science substudy, and it will be marked on the consent form as optional. The sample size for the substudy is 200 patients.

10.1.1. Procedure

Participants will have four or five blood samples taken; blood samples are taken pre-operatively, and postoperatively on days 1, 3 and 5. If the participant attends a clinical

follow-up visit (at 6 weeks +/- one month) then a further blood sample will be taken. Approximately 10ml of blood is required for each sample.

The blood samples will usually be taken at the same time as routine clinical samples and, where possible, post-operative blood samples will be drawn from existing intravascular lines. Additional venepuncture may however be required in some cases.

10.1.2. Sample processing and storage

The blood samples will be assigned a unique pseudonymised identifier linked to the Tight K study number. No other identifiable information will be stored with the samples.
All blood samples will be received, tracked, processed and stored by the Tight K Basic Science team based at the William Harvey Research Institute.

10.2. Data Science substudy

Machine learning and artificial intelligence analytics will be used to analyse anonymised eCRF data and trial cardiographic data in order to identify factors that predict the onset of AF. These insights will inform the development of interventions to prevent AF.

11. Monitoring and Audits

The conduct of the trial will be supervised by trained staff from the LSHTM CTU. The trial will be monitored on a regular basis using central statistical monitoring. Full details will be available in the monitoring working practice document (WPD) and the trial will be monitored according to this agreed plan.

Local investigators shall ensure that all trial data are available for trial-related monitoring, and sponsor and regulatory authority audits. The sponsor also holds the right to monitor or audit the study.

12. Safety monitoring

12.1. Definition

Safety reporting for each patient should commence from time of randomisation to completion of follow-up at 6 months after the CABG surgery. Pre-existing conditions that are unchanged after the trial treatment period should not be reported as adverse events. For any participants who do not have CABG surgery safety reporting will commence at randomisation and complete 6 months after the randomisation date.

Expected events are defined as any of the outcomes (see Section 6.4.1, 6.5.2) or expected complications of potassium supplementation, CABG surgery or usual clinical care (see Section 12.2). These events may or may not require hospitalisation or prolongation of existing hospitalisation. These events will be recorded in the eCRF, but do not need to be separately reported.

Unexpected events are defined as all other events and should be reported as either a serious adverse event (SAE) or non-serious adverse event (NSAE), depending on their severity.

12.2. Expected adverse events

- Skin irritation from ECG electrodes
- Hyperkalaemia ($[K^+]$ ≥5.5 mEq/L)
- Line site complications (phlebitis, infection etc.)
- Nausea
- Constipation*
- Vomiting*
- Myocardial infarction*
- Stroke
- Renal failure requiring dialysis*
- Renal impairment not requiring dialysis*
- Wound infection (sternum or donor site)*
- Return to theatre for bleeding*
- Prolonged mechanical support
- Post-op delirium
- Non-cardiac chest pain*
- Heart failure*
- Pleural effusion*
- Chest drain insertion*
- Pericardial effusion*
- Chest infection (pneumonia)*
- Lung atelectasis*
- Pneumothorax*
- Shortness of breath caused by any expected AEs marked above with an asterisk(*)
- Blood transfusion
- Pericarditis
- Ulnar nerve paraesthesia
- Heart block requiring pacemaker
- Urinary tract infection
- Suprapubic catheter
- Urinary retention
- Intra-aortic balloon pump insertion

12.3. Unexpected Serious Adverse Events

Any untoward medical occurrence/effect that:

1. Results in death
2. Is life-threatening*
3. Requires hospitalisation or prolongation of existing hospitalisation
4. Results in persistent or significant disability or incapacity
5. Consists of a congenital anomaly or birth defect
6. Is otherwise considered medically significant by the investigator

*Life-threatening, in the definition of a SAE, refers to an event in which the patient was at risk of death at the time of event. It does not refer to an event that hypothetically might have caused death if it were more severe.

Unexpected SAEs should be reported to the CTU within 7 days of the site becoming aware of the event. The report should include an assessment of seriousness and causality (see Section 12.5.2) by the Principal Investigator, or a member of staff delegated this task, at each site.

The Chief Investigator will be responsible for the prompt notification of findings that could adversely affect the health of patients or impact on the conduct of the trial. The Sponsor, the Research Ethics Committee (REC) and the Data Monitoring Committee (DMC) will be notified by the CTU when reported SAEs have been classified by the Chief Investigator as both unexpected and given a causality classification of either Probable or Possible.

12.4. Unexpected Non-Serious Adverse Events

Unexpected NSAEs should be evaluated by the PI or a member of staff delegated this task. This should include an assessment of causality (see Section 12.5.2) and intensity (see Section 12.5.1) and reports made within 14 days of the site becoming aware of the event. The CTU will keep detailed records of all unexpected adverse events reported. Reports will be reviewed by the Chief Investigator to consider intensity, causality and expectedness. As appropriate, these will be reported to the Sponsor, the DSMC and the REC.

12.5. Reporting unexpected adverse events

Investigators will make their reports of all unexpected adverse events, whether serious or not, to the CTU at LSHTM.

12.5.1. Assessment of intensity

Mild: The patient is aware of the event or symptom, but the event or symptom is easily tolerated.
Moderate: The patient experiences sufficient discomfort to interfere with or reduce his or her usual level of activity.
Severe: Significant impairment of functioning; the patient is unable to carry out usual activities and/or the patient's life is at risk from the event.

12.5.2. Assessment of causality

Probable: A causal relationship is clinically / biologically highly plausible and there is a plausible time sequence between onset of the adverse event and being on the trial.
Possible: A causal relationship is clinically / biologically plausible and there is a plausible time sequence between onset of the adverse event and being on the trial.
Unlikely: A causal relationship is improbable and another documented cause of the adverse event is most plausible.
Unrelated: A causal relationship can definitely be excluded and another documented cause of the adverse event is most plausible.

Events that are classified as either probable or possible will be treated as related for the purposes of AE reporting.

12.6. Urgent Safety Measures

The Chief Investigator may take urgent safety measures to ensure the safety and protection of the clinical trial patients from any immediate hazard to their health and safety. The measures should be taken immediately. In this instance, the approval of the REC prior to implementing these safety measures is not required. However, it is the responsibility of the Chief Investigator to inform the Sponsor and REC (via telephone) of this event immediately.

The Chief Investigator has an obligation to inform the REC in writing within 3 days, in the form of a substantial amendment. The Sponsor (Joint Research Management Office [JRMO]) must be sent a copy of the correspondence.

12.7. Annual Safety Reporting

The Chief Investigator will send an Annual Progress Report to the main REC using their template (the anniversary date is the date on the REC “favourable opinion” letter from the REC) and to the Sponsor.

12.8. Overview of the Safety Reporting responsibilities

The Chief Investigator has the overall safety oversight responsibility. The Chief Investigator has a duty to ensure that safety monitoring and reporting is conducted in accordance with the Sponsor’s requirements.

13. Statistical Considerations

13.1. Power calculations and sample size determination

1684 participants are to be recruited from 15-25 centres allocated to each of the trial arms in a ratio of 1:1.

The sample-size calculation is based on an incidence of new onset AFACS (atrial fibrillation, flutter and/or tachyarrhythmia) in the tight potassium control arm of 35%, which is at the lower end of the published figures and supported by our pilot data that showed an overall AFACS incidence of 36.9% (95% confidence interval [CI] 29.1 to 44.9).

The co-applicants (from diverse backgrounds in cardiothoracic surgery, cardiothoracic intensive care, cardiology and clinical trial management) reached consensus that a clinically relevant non-inferiority margin is 10%. If there is a true difference in favour of tight potassium control of 2%, then 1514 participants are required to be 90% certain that the upper limit of a one-sided 97.5% CI (or equivalently a 95% two-sided CI) will exclude a difference in favour of tight potassium control of more than 10%. Allowing for a 10% loss to follow-up means we need to recruit 1684 participants.

13.2. Trial statistician

The trial statistician is Professor Elizabeth Allen. Statistical analysis will be coordinated from the CTU at LSHTM.

13.3. Statistical analysis

Statistical analysis of outcomes will be carried out blind to treatment allocation.

13.3.1. Summary of baseline data and flow of participants

Baseline characteristics of enrolled participants will be summarised by treatment arm. Descriptive statistics for continuous variables will include mean, standard deviation, median, range and number of observations. Categorical variables will be summarised as counts and proportions. Screening, enrolment, reasons for non-enrolment, randomisation and loss to follow-up will be detailed in a CONSORT flowchart.

13.3.2. Primary and secondary outcome analyses

The primary and secondary efficacy outcome analysis will be carried out using an efficacy analysis population which excludes patients who did not undergo isolated CABG. An analysis on a per-protocol population will also be carried out. Definition of the per-protocol analysis will be determined before database lock.

Analysis will follow a pre-specified analysis plan approved by the senior statistician, Chief Investigator and the DSMB prior to unblinding the study database. Every effort will be made to obtain outcome measures on all participants. No significance tests will be performed to test for differences at baseline. The primary analysis will be an unadjusted analysis.

For the primary outcome(incidence of new onset AFACS until hour 120 after surgery or discharge from hospital if sooner), we will use a one-sided 97.5 confidence interval approach to test for non-inferiority between the two treatments.

The one-sided 97.5% CI for the between group difference point estimate will be calculated. Non-inferiority of the relaxed arm will be accepted if the upper bound of the 97.5% CI lies within the pre-specified non-inferiority margin of 10%.

The same approach will be used for the secondary outcomes; inpatient and six-month mortality after enrolment (secondary outcomes).

For all other secondary outcomes, we will use linear regression to test for non-inferiority between the two arms of the trial. Effect sizes between treatment conditions will be calculated and following convention (Cohen 1988) statistically significant effect sizes <=0.2 will be considered not relevant and for this study a non-inferior difference between conditions.

An adjusted analysis will be carried out, adjusted for the stratification factor site with any other adjustment factors pre-specified in the Statistical analysis plan. Additional exploratory analyses will control for any baseline measures that appear to be imbalanced between arms. All subgroup analyses will be specified a priori in the Statistical Analysis Plan and carried out using formal tests for interaction included in the statistical models and assessed for statistical significance using Likelihood ratio tests.

13.3.3. Exploratory analyses

The following outcomes will be investigated in the exploratory analyses:

- Duration of Holter-identified AFACS. This will be the mean of the following for each patient; total time spent in AF divided by the time the monitor was attached. This will be identified on Holter monitors until hour 120 after initial admission to ICU/post-operative care facility or discharge from hospital, whichever occurs first.
- Median number of Holter-identified AFACS episodes experienced by patients until hour 120 after initial admission to ICU/post-operative care facility or discharge from hospital, whichever occurs first.

14. Ethics

14.1. Declaration of Helsinki and Good Clinical Practice

The study will conform to the spirit and the letter of the Declaration of Helsinki, and in accordance with Barts Health and ICH-GCP. The study will be carried out in accordance with the ethical principles in the Research Governance Framework for Health and Social Care, Second Edition, 2005 and its subsequent amendments as applicable and applicable legal and regulatory requirements.

14.2. UK ethics committee review

Health Research Authority (HRA) Research Ethics Committee London – Queen's Square have reviewed and approved the trial for sites in the UK. The REC number is 19/LO/1064. Each centre will retain a copy of the approval letter in the trial site file.

14.3. Confidentiality Advisory Group

Permission has been granted by the Confidentiality Advisory Group (CAG) to allow hospital notes at sites in the UK to be screened for eligibility by delegated members of the research team without needing written consent, and for staff to contact patients prior to a clinic appointment to inform them about the trial. The CAG reference number is 19/CAG/0146.

15. Management and oversight

15.1. Trial Management Group (TMG)

Prof Ben O'Brien (St Bartholomew's Hospital)
Prof Julie Sanders (St Bartholomew's Hospital)
Mr Neil Roberts (St Bartholomew's Hospital)
Dr Niall Campbell (Wythenshawe Hospital)
Prof Hugh Montgomery (University College London)
Ms Trudie Lobban (Arrhythmia Alliance)
Prof Diana Elbourne (LSHTM)
Prof Elizabeth Allen (LSHTM)
Dr Charles Opondo (LSHTM)
Mr Richard Evans (LSHTM)
Ms Laura Van Dyck (LSHTM)

Ms Zahra Jamal (LSHTM)

15.2. Trial Steering Committee (TSC)

Dr Rob Henderson (Trent Cardiac Centre) – Chair
Mr Jatin Desai (King's College Hospital, retired) – Independent
Prof David Keane (St Vincent's University Hospital, Dublin) - Independent
Dr Kurt Rützler (Cleveland Clinic, USA) – Independent
Mr Steve Stevenson (Patient representative) – Independent
Mr Richard Duncker (Patient representative) – Independent
Mrs Rosalie Renamagboo (Barts Health NHS Trust) – Non-independent
Dr Nick Barrett (St Thomas' Hospital) – Non-independent
Prof Ben O'Brien (St Bartholomew's Hospital) – Chief Investigator
Prof Diana Elbourne (LSHTM) – Co-investigator

15.3. Data Safety and Monitoring Committee (DSMC)

Dr Philip Jones (University of Western Ontario, Canada) – Chair
Dr Ly-Mee Yu (University of Oxford)
Dr Bob Kiaii (UC Davis Medical Centre, USA)
Ms Joanna Sturgess (LSHTM) will be the statistician reporting to the DSMC.

15.4. Event Validation Committee (EVC)

Prof Gavin Murphy (Glenfield Hospital) – Chair
Other members to be confirmed.

16. Finance and Funding

This trial is funded by the British Heart Foundation (BHF).

17. Indemnity

17.1. Sponsorship

This trial is sponsored by Barts Health NHS Trust.

17.2. Insurance

All recruiting centres in the UK will be covered by NHS indemnity for negligent harm providing researchers hold a contract of employment with the NHS, including honorary contracts held by academic staff. Medical co-investigators will also be covered by their own medical defence insurance for non-negligent harm.

18. Dissemination of Research Findings

It is our intention to disseminate the results of the trial as widely as possible, including to the patients who participated. This is likely to be through a publication in a peer reviewed journal. Publications will follow the CONSORT guidelines[25]. Authorship will follow international guidelines.

19. References

1. Maisel WH, Rawn JD, Stevenson WG. Atrial fibrillation after cardiac surgery. Annals of internal medicine. 2001 Dec 18;135(12):1061-73
2. Mathew JP, Fontes ML, Tudor IC, Ramsay J, Duke P, Mazer CD, et al. A multicenter risk index for atrial fibrillation after cardiac surgery. Jama. 2004 Apr 14;291(14):1720-9
3. Helgadottir S, Sigurdsson MI, Ingvarsdottir IL, Arnar DO, Gudbjartsson T. Atrial fibrillation following cardiac surgery: risk analysis and long-term survival. Journal of cardiothoracic surgery. 2012 Sep 19;7:87
4. Villareal RP, Hariharan R, Liu BC, Kar B, Lee VV, Elayda M, et al. Postoperative atrial fibrillation and mortality after coronary artery bypass surgery. J Am Coll Cardiol. 2004 Mar 3;43(5):742-8
5. Mariscalco G, Klersy C, Zanobini M, Banach M, Ferrarese S, Borsani P, et al. Atrial fibrillation after isolated coronary surgery affects late survival. Circulation. 2008 Oct 14;118(16):1612-8
6. El-Chami MF, Kilgo P, Thourani V, Lattouf OM, Delurgio DB, Guyton RA, et al. New-onset atrial fibrillation predicts long-term mortality after coronary artery bypass graft. J Am Coll Cardiol. 2010 Mar 30;55(13):1370-6
7. Sanders J, Keogh BE, Van der Meulen J, Browne JP, Treasure T, Mythen MG, et al. The development of a postoperative morbidity score to assess total morbidity burden after cardiac surgery. Journal of clinical epidemiology. 2012;65(4):423-33
8. Aranki SF, Shaw DP, Adams DH, Rizzo RJ, Couper GS, VanderVliet M, et al. Predictors of atrial fibrillation after coronary artery surgery. Current trends and impact on hospital resources. Circulation. 1996 Aug 1;94(3):390-7
9. Zimmer J, Pezzullo J, Choucair W, Southard J, Kokkinos P, Karasik P, et al. Meta-analysis of antiarrhythmic therapy in the prevention of postoperative atrial fibrillation and the effect on hospital length of stay, costs, cerebrovascular accidents, and mortality in patients undergoing cardiac surgery. The American journal of cardiology. 2003;91(9):1137-40
10. Borzak S, Tisdale JE, Amin NB, Goldberg AD, Frank D, Padhi ID, et al. Atrial fibrillation after bypass surgery: does the arrhythmia or the characteristics of the patients prolong hospital stay? Chest. 1998 Jun;113(6):1489-91
11. Schnabel RB, Sullivan LM, Levy D, Pencina MJ, Massaro JM, D'Agostino RB, Sr., et al. Development of a risk score for atrial fibrillation (Framingham Heart Study): a community-based cohort study. Lancet (London, England). 2009 Feb 28;373(9665):739-45
12. O'Brien B, Burrage PS, Ngai JY, Prutkin JM, Huang CC, Xu X, et al. Society of Cardiovascular Anesthesiologists/European Association of Cardiothoracic Anaesthetists Practice Advisory for the Management of Perioperative Atrial Fibrillation in Patients Undergoing Cardiac Surgery. Journal of cardiothoracic and vascular anesthesia. 2019 Jan;33(1):12-26
13. Muehlschlegel JD, Burrage PS, Ngai JY, Prutkin JM, Huang CC, Xu X, et al. Society of Cardiovascular Anesthesiologists/European Association of Cardiothoracic Anaesthetists Practice Advisory for the Management of Perioperative Atrial Fibrillation in Patients Undergoing Cardiac Surgery. Anesthesia and analgesia. 2019 Jan;128(1):33-42
14. Podrid PJ. Potassium and ventricular arrhythmias. Am J Cardiol. 1990 Mar 6;65(10):33E-44E; discussion 52E

15. Polderman KH, Girbes AR. Severe electrolyte disorders following cardiac surgery: a prospective controlled observational study. Crit Care. 2004 Dec;8(6):R459-66
16. Krijthe BP, Heeringa J, Kors JA, Hofman A, Franco OH, Witteman JC, et al. Serum potassium levels and the risk of atrial fibrillation: the Rotterdam Study. International journal of cardiology. 2013 Oct 15;168(6):5411-5
17. Dunning J, Treasure T, Versteegh M, Nashef SA. Guidelines on the prevention and management of de novo atrial fibrillation after cardiac and thoracic surgery. European journal of cardio-thoracic surgery : official journal of the European Association for Cardio-thoracic Surgery. 2006 Dec;30(6):852-72
18. Arsenault KA, Yusuf AM, Crystal E, Healey JS, Morillo CA, Nair GM, et al. Interventions for preventing post-operative atrial fibrillation in patients undergoing heart surgery. Cochrane Database of Systematic Reviews [Internet]. 2013; (1). Available from: http://onlinelibrary.wiley.com/doi/10.1002/14651858.CD003611.pub3/abstract.
19. Weiner ID, Wingo CS. Hypokalemia--consequences, causes, and correction. Journal of the American Society of Nephrology : JASN. 1997 Jul;8(7):1179-88
20. Pittet D, Tarara D, Wenzel RP. Nosocomial bloodstream infection in critically ill patients. Excess length of stay, extra costs, and attributable mortality. Jama. 1994 May 25;271(20):1598-601
21. Norris W, Kunzelman KS, Bussell S, Rohweder L, Cochran RP. Potassium Supplementation, Diet vs Pills*. Chest. 2004 February 1, 2004;125(2):404-9
22. Cohn JN, Kowey PR, Whelton PK, Prisant LM. New Guidelines for Potassium Replacement in Clinical Practice: A Contemporary Review by the National Council on Potassium in Clinical Practice. Arch Int Med2000. p. 2429-36.
23. Pilot Study for the Tight K Study (Tight K) [14/02/2019]. Available from: https://clinicaltrials.gov/ct2/show/NCT03195647.
24. Calkins H, Kuck KH, Cappato R, Brugada J, Camm AJ, Chen SA, et al. 2012 HRS/EHRA/ECAS expert consensus statement on catheter and surgical ablation of atrial fibrillation: recommendations for patient selection, procedural techniques, patient management and follow-up, definitions, endpoints, and research trial design: a report of the Heart Rhythm Society (HRS) Task Force on Catheter and Surgical Ablation of Atrial Fibrillation. Developed in partnership with the European Heart Rhythm Association (EHRA), a registered branch of the European Society of Cardiology (ESC) and the European Cardiac Arrhythmia Society (ECAS); and in collaboration with the American College of Cardiology (ACC), American Heart Association (AHA), the Asia Pacific Heart Rhythm Society (APHRS), and the Society of Thoracic Surgeons (STS). Endorsed by the governing bodies of the American College of Cardiology Foundation, the American Heart Association, the European Cardiac Arrhythmia Society, the European Heart Rhythm Association, the Society of Thoracic Surgeons, the Asia Pacific Heart Rhythm Society, and the Heart Rhythm Society. Heart rhythm : the official journal of the Heart Rhythm Society. 2012 Apr;9(4):632-96 e21
25. Piaggio G, Elbourne DR, Pocock SJ, Evans SJW, Altman DG, for the CONSORT Group. Reporting of noninferiority and equivalence randomized trials. Extension of the CONSORT 2010 statement. JAMA. 2012; 308(24): 2594-2604. doi:10.1001/jama.2012.87802. PMID: 23268518

Appendix 1: Amendment History

Amendment No.	Protocol Version No.	Date issued	Author(s) of changes	Details of Changes made
2	2.0	02.11.2021	Kimberley Potter / Richard Evans	• Clarification of terminology used<br>o AF (Atrial Fibrillation) has been updated to AFACS (Atrial Fibrillation After Cardiac Surgery).<br>o AFACS definition has been included to clearly incorporate atrial fibrillation, atrial flutter and atrial tachycardia.<br>• Re-wording of the primary and secondary outcomes to make them clearer and less open to misinterpretation.<br>• Minor clarifications to the inclusion and exclusion criteria to make them easier for sites to understand and apply appropriately.<br>• Removal of Tight K Australia as a partner trial due to their funding application being unsuccessful.<br>• Added definitions of Holter-identified AF and non-AF arrhythmias.<br>• Information relating to independent adjudication of the primary outcome has been added. This is to ensure that the primary endpoint is less open to potential bias.<br>• Procedures for withdrawing from follow up procedures have been added, to ensure that patients who wish to withdraw have clearer options.<br>• Procedures for CABG postponement or cancellation post-randomisation have been added to make this as clear as possible for sites.<br>• Information about the Holter monitoring procedure have been added.

				• Clarification of the data collected in the CRF and improvements to the data collection process have been made.<br>• Clarification of the data collected on adverse events has been added.
3	3.0	01.02.2023	Zahra Jamal	• Clarified in the primary outcome that AFACS needs to be clinically detected and electrocardiographically confirmed.<br>• Trial secondary outcomes updated.<br>• Extended the duration of the trial to 63 months.<br>• Revised process for event validation of the primary outcome<br>• Addition of participating self-sponsored sites in Germany<br>• Clarified the timing of randomisation.<br>• The definition of isolated CABG surgery (section 7.3) has been added to provide clarification on the eligibility criteria<br>• Addition of the Basic Science and Data Science substudies.<br>• Clarified that pre-existing conditions do not need to be reported as AEs.<br>• Updated Trial Management Group, Trial Steering Committee, Data Monitoring Committee and Event Validation Committee membership
