# Supplementary material for: Impact of maintaining serum potassium concentration ≥ 3.6mEq/L versus ≥ 4.5mEq/L for 120 hours after isolated coronary artery bypass graft surgery on incidence of new onset atrial fibrillation: Protocol for a randomized non-inferiority trial
Source: PLoS One. 2024 Mar 13;19(3):e0296525. doi: 10.1371/journal.pone.0296525 (PMC10936833; doi:10.1371/journal.pone.0296525)
Supplement: S4 File — (PDF) [file pone.0296525.s004.pdf]

[redacted]

TIGHT K

Prevention of dysrhythmias on the cardiac intensive care unit - does maintenance of high-normal serum potassium levels matter?

Tight K Trial Protocol

Version 1 26/04/2019

Funded by the British Heart Foundation

Sponsored by Barts Health NHS Trust

Managed by London School of Hygiene & Tropical Medicine Clinical Trials Unit

**Full Title**	The TIGHT-K STUDY. Prevention of dysrhythmias on the cardiac intensive care unit - does maintenance of high-normal serum potassium levels matter?
**Short Title/Acronym**	Tight K Trial
**Sponsor**	Barts Health NHS Trust<br>Dr Mays Jawad<br>Director of Research Services<br>Joint Research Management Office<br>5 Walden Street<br>London<br>E1 2EF<br>[redacted]
**Sponsor trial number**	*TO BE ADDED*
**REC Reference**	*TO BE ADDED*
**IRAS Reference**	260639
**Chief Investigator**	Prof Ben O'Brien<br>Professor of Perioperative Medicine<br>Consultant in Intensive Care Medicine and Cardiac Anaesthesia<br>Barts Health NHS Trust<br>St Bartholomew's Hospital<br>London<br>EC1A 7DN<br>[redacted]
**Clinical Trials Unit**	Department of Medical Statistics<br>London School of Hygiene & Tropical Medicine<br>Keppel Street<br>London<br>WC1E 7HT<br>[redacted]

Contents

1. **GLOSSARY OF TERMS AND ABBREVIATIONS** **5**
2. **SIGNATURE PAGE** **6**
   2.1. CHIEF INVESTIGATOR AGREEMENT 6
   2.2. STATISTICIAN AGREEMENT 6
3. **SUMMARY / SYNOPSIS** **7**
   3.1. PROTOCOL SUMMARY 7
   3.2. TRIAL FLOWCHART 9
4. **INTRODUCTION** **10**
   4.1. BACKGROUND 10
   4.2. FEASIBILITY STUDY RESULTS 11
   4.3. RATIONALE FOR THE TRIAL 11
5. **AIM AND OBJECTIVES** **12**
   5.1. AIM 12
   5.2. HYPOTHESIS 12
   5.3. OBJECTIVES 12
      5.3.1. Primary objective 12
      5.3.2. Secondary objectives 12
6. **TRIAL SETTING AND DESIGN** **13**
   6.1. SETTING 13
      6.1.1. Site requirements 13
      6.1.2. Site and PI responsibilities 13
      6.1.3. Site initiation and activation 13
   6.2. TRIAL DESIGN 14
      6.2.1. Primary outcome 14
      6.2.2. Secondary outcomes 14
7. **SELECTION AND WITHDRAWAL OF PARTICIPANTS** **15**
   7.1. INCLUSION CRITERIA 15
   7.2. EXCLUSION CRITERIA 15
   7.3. CO-ENROLMENT 15
   7.4. WITHDRAWAL OF PARTICIPANTS 15
      7.4.1. Criteria for withdrawal from the trial 15
      7.4.2. Withdrawing from the trial treatment 15
      7.4.3. Withdrawing from the trial 15
      7.4.4. Reporting withdrawal 16
8. **TRIAL PROCEDURES** **16**
   8.1. SCREENING 16
      8.1.1. Screening log 16
   8.2. INFORMED CONSENT PROCEDURE 16
   8.3. RANDOMISATION 16
      8.3.1. Intervention arm 17
      8.3.2. Control arm 17
      8.3.3. Blinding 17
   8.4. TRIAL TREATMENT 17
      8.4.1. Patients with AF 17
   8.5. HOLTER MONITORING 17
   8.6. FOLLOW-UP 18
   8.7. END OF TRIAL 18

9. DATA COLLECTION 19
9.1. TRIAL TREATMENT PERIOD 19
9.2. TRIAL PROCEDURES TABLE 19
9.3. DATA COLLECTION 19
9.4. TRIAL PROCEDURES 20
9.4.1. *Before surgery* *20*
9.4.2. *Baseline* *20*
9.4.3. *Period 1-5* *20*
9.4.4. *Discharge (from ICU and hospital)* *20*
9.4.5. *Follow-up* *21*
9.5. COMPLIANCE AND LOSS TO FOLLOW-UP 21
9.5.1. *Loss to follow-up* *21*
9.5.2. *Compliance* *21*
9.6. DATA HANDLING AND RECORD KEEPING 21
10. MONITORING AND AUDITS 22
11. SAFETY MONITORING 22
11.1. DEFINITION 22
11.2. EXPECTED ADVERSE EVENTS 22
11.3. UNEXPECTED SERIOUS ADVERSE EVENTS 23
11.4. UNEXPECTED NON-SERIOUS ADVERSE EVENTS 23
11.5. REPORTING UNEXPECTED ADVERSE EVENTS 23
11.5.1. *Assessment of intensity* *23*
11.5.2. *Assessment of causality* *24*
11.6. URGENT SAFETY MEASURES 24
11.7. ANNUAL SAFETY REPORTING 24
11.8. OVERVIEW OF THE SAFETY REPORTING RESPONSIBILITIES 24
12. STATISTICAL CONSIDERATIONS 25
12.1. POWER CALCULATIONS AND SAMPLE SIZE DETERMINATION 25
12.1.1. *Tight K Australia* *25*
12.2. TRIAL STATISTICIAN 25
12.3. STATISTICAL ANALYSIS 25
12.3.1. *Summary of baseline data and flow of participants* *25*
12.3.2. *Primary and secondary outcome analyses* *25*
13. ETHICS 26
13.1. DECLARATION OF HELSINKI AND GOOD CLINICAL PRACTICE 26
13.2. ETHICAL COMMITTEE REVIEW 26
13.3. CONFIDENTIALITY ADVISORY GROUP 26
14. MANAGEMENT AND OVERSIGHT 27
14.1. TRIAL MANAGEMENT GROUP (TMG) 27
14.2. TRIAL STEERING COMMITTEE (TSC) 27
14.3. DATA SAFETY AND MONITORING COMMITTEE (DSMC) 27
15. FINANCE AND FUNDING 27
16. INDEMNITY 27
16.1. SPONSORSHIP 27
16.2. INSURANCE 27
17. DISSEMINATION OF RESEARCH FINDINGS 28
18. REFERENCES 29

1. Glossary of Terms and Abbreviations

AE	adverse event
AF	atrial fibrillation
AV	atrioventricular
BHF	British Heart Foundation
CABG	coronary artery bypass graft
CAG	Confidentiality Advisory Group
CI	confidence interval
CRF	case report form
CTU	Clinical Trials Unit
DSMC	Data Safety and Monitoring Committee
ECG	electrocardiogram
eCRF	electronic case report form
EQ-5D-5L	EuroQol EQ-5D 5-level questionnaire
HRA	Health Research Authority
ICH-GCP	International Council for Harmonisation Good Clinical Practice
ICU	Intensive Care Unit
IRAS	Integrated Research Application System
ITT	Intention-to-treat
IV	intravenous
JRMO	Joint Research Management Office
[K+]	potassium concentration
LSHTM	London School of Hygiene & Tropical Medicine
NHS	National Health Service
NSAE	non-serious adverse event
Participant	An individual who takes part in a clinical trial
PI	Principal Investigator
PIS	Participant Information Sheet
RCT	randomised clinical trial
REC	Research Ethics Committee
SAE	serious adverse event
SOP	standard operating procedure
TMG	Trial Management Group
TSC	Trial Steering Committee

2. Signature Page

2.1. Chief Investigator Agreement

The clinical study as detailed within this research protocol **(Version 1, 26/04/2019)**, or any subsequent amendments will be conducted in accordance with the current Research Governance Framework for Health & Social Care (2005), the World Medical Association Declaration of Helsinki (1996) and the current applicable regulatory requirements and any subsequent amendments of the appropriate regulations.

Chief Investigator Name: Prof Ben O'Brien

Chief Investigator Site: Barts Heart Centre

Signature: [redacted] **Date:** 26/04/2019

2.2. Statistician Agreement

The clinical study as detailed within this research protocol **(Version 1, 26/04/2019)** or any subsequent amendments will be conducted in accordance with the current Research Governance Framework for Health & Social Care, the World Medical Association Declaration of Helsinki (1996), Principles of ICH E6-GCP, ICH E9 - Statistical principles for Clinical Trials, ICH E10 - Choice of Control Groups and the current applicable regulatory requirements and any subsequent amendments of the appropriate regulations.

Statistician Name: Professor Elizabeth Allen

Statistician Site: London School of Hygiene and Tropical Medicine

Signature: [redacted] **Date:** 26/04/2019

3. Summary / Synopsis

3.1. Protocol Summary

Short Title	Tight K Trial
Design	Multicentre, non-inferiority randomised clinical trial
Sites	15-25 NHS Hospitals
Aim	To determine whether maintaining serum potassium levels at ≥3.6 mEq/L is non-inferior to normal treatment (≥4.5 mEq/L) on the occurrence of new onset atrial fibrillation (AF) dysrhythmia post-surgery in patients undergoing isolated coronary artery bypass graft (CABG) surgery
Primary outcome	The presence of new onset AF until hour 120 after surgery or discharge from hospital, whichever is sooner
Secondary outcomes	• Prevalence of all AF including those identified on holter monitors<br>• Prevalence of all other non-AF dysrhythmias, defined using standard diagnostic criteria<br>• In-patient mortality<br>• 6-month mortality<br>• Critical care length of stay<br>• Hospital length of stay<br>• Costs relating to potassium therapy<br>• Quality of life at 6 months
Inclusion criteria	Undergoing isolated CABG surgery
Exclusion criteria	• Age <18<br>• Previous AF<br>• Ongoing infection/sepsis at the time of recruitment<br>• Pre-operative high-degree atrioventricular block<br>• Pre-operative serum K+ >5.5 mEq/L<br>• Current/previous use of medication for the purposes of cardiac rhythm management<br>• Dialysis-dependent end-stage renal failure<br>• Concurrent patient involvement in another clinical trial assessing cardiac rhythm post-operative interventions<br>• Unable to give informed consent
Number of Participants	Tight K Trial (UK): 1684 participants, approx. 842 in each trial arm<br><br>This trial is a collaboration with a partner trial in Australia (Tight K), which will recruit 550 participants.<br><br>In total, the number of participants is 2234

Trial arms	Intervention: Serum potassium levels maintained at ≥3.6 mEq/L ('Relaxed')<br><br>Control: Serum potassium levels maintained at ≥4.5 mEq/L ('Tight')
Trial duration	4.5 years

3.2. Trial Flowchart

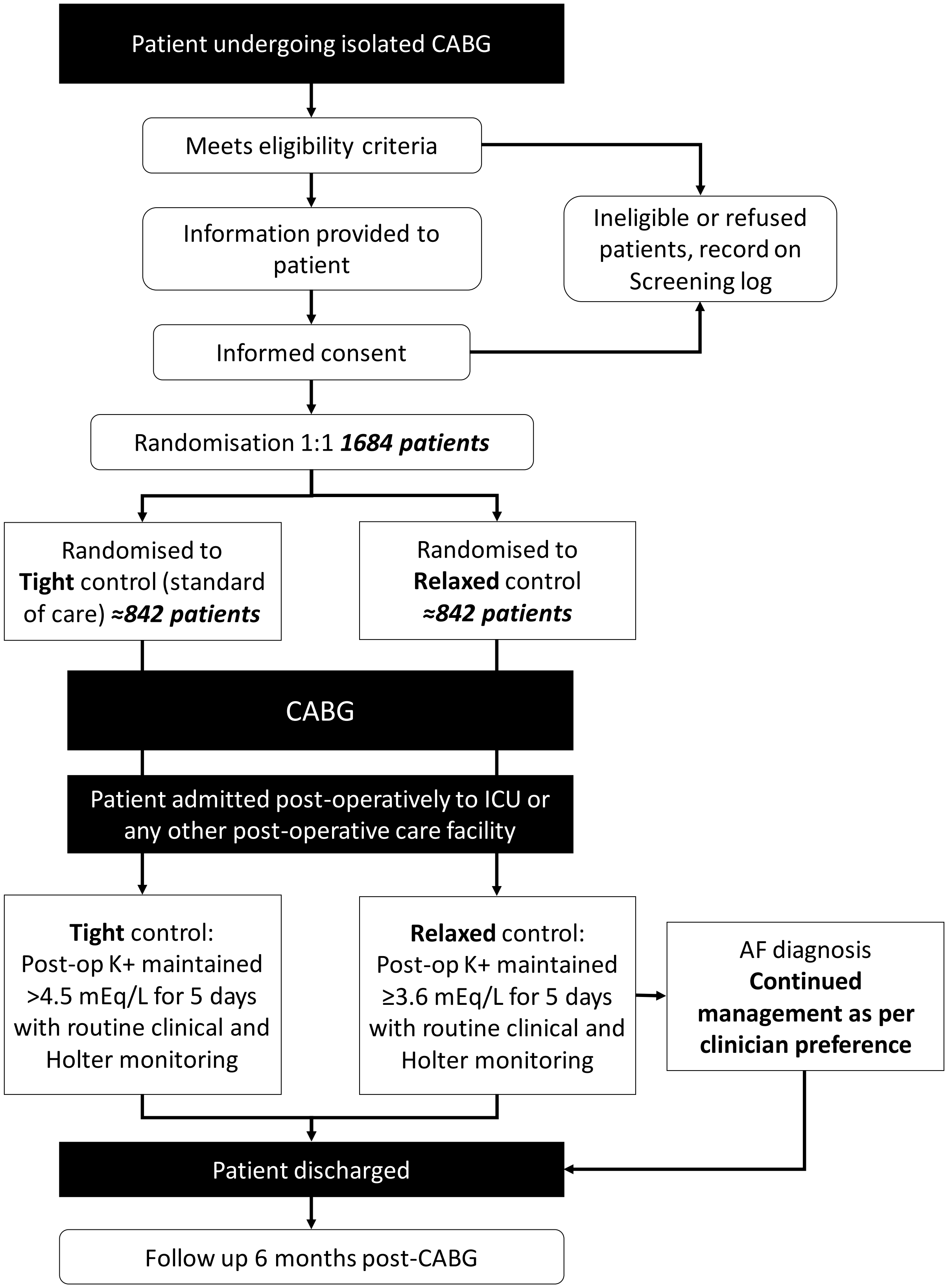

4. Introduction

4.1. Background

At least one in three patients is affected by atrial fibrillation (AF) after cardiac surgery, with most episodes occurring in the first five postoperative days[1-3]. AF occurrence is associated with increased morbidity, short and long-term mortality[3-6], intensive care unit (ICU) and hospital stay[7, 8], and cost of care[9]. Persistence of these associations after adjustment for potential confounding factors suggests that they may be causal[10]. The incidence and prevalence of AF and its associated costs are expected to increase as the surgical population ages[11]. Extensive effort is undertaken to prevent AF after cardiac surgery from occurring, but clinical practice in this area is highly variable and the evidence base for most interventions is sparse[12, 13].

Potassium plays an important role in cardiac electrophysiology[14]. Serum potassium concentrations ([K+]) are commonly low following cardiac surgery[15], and appear marginally lower in those suffering atrial arrhythmias in non-surgical cohorts[16]. Despite an absence of proof that this association is causal, efforts to maintain serum [K+] in the 'high-normal' range (≥ 4.5 mEq/L), as opposed to just intervening if potassium drops below its lower 'normal' threshold (<3.6 mEq/L), are considered 'routine practice' for AF prevention in post-surgical patients in many centres across the world[17]. From the (unpublished) data from our British Heart Foundation (BHF) funded Tight-K Feasibility Study, all 160 patients would have required at least one dose of potassium to supplement their levels to this high-normal range and 45.5% of all serum [K+] measurements were below 4.5mEq/L at some point. Data from the same pilot study show a median number of potassium doses given in the 'tight' group (high-normal serum potassium target) of seven, compared to a median of one, with most patients not receiving any potassium supplementation at all, in the 'relaxed' group. We did, for the first time ever, show that the practice does achieve a separation in serum potassium levels between the two groups, so the protocol is indeed effective in achieving higher serum potassium levels.

The efficacy of the practice of maintaining high-normal serum potassium levels for the prevention of AF after cardiac surgery, however, remains unproven and data supporting it are extremely limited, being derived from observational studies rather than randomised trials[17]. Indeed, no data exist to demonstrate that maintaining a high-normal potassium level is beneficial in these circumstances, or that aggressive replenishment of potassium in these patients improves outcome[18].

Meanwhile, potassium supplementation may cause discomfort or harm. Routine central venous potassium administration in the early post-operative period, when oral supplementation is not possible, is time-consuming, costly and associated with clinical risk: rapid infusion can prove fatal[19], and leaving central venous catheters in situ for the sole purpose of potassium replacement increases infection risk[20]. Oral replacement (when feasible) is commonly associated with profound nausea and gastrointestinal side effects, and is very poorly tolerated by patients[21, 22]. The annual costs of intravenous potassium exceed those for other drugs in many cardiac surgical units due to the large quantities administered[23]. Nursing time (e.g. for drug checks and administration) will add to this cost.

4.2. Feasibility study results

We recruited 160 patients between 28 August 2017 and 24 April 2018. The average recruitment rate was thus 20 patients per month over two sites. Of 601 screened patients, 24% were recruited, 48% were eligible but not recruited and 27% were ineligible. Randomisation was acceptable and was successful in all recruited patients. Provisional data on the need for potassium administration showed that in the relaxed arm, 19 out of 79 patients (24.1%) had at least one measurement below 3.6mEq/L and therefore required potassium supplementation. In the tight arm, 80 out of 81 patients (98.8%) with potassium data had at least one measurement below 4.5mEq/L and required potassium. Data available on potassium protocol violations demonstrated a rate of 9.8% (283/2886) based on the total number of violations/total number of potassium measurements.

Follow-up rates were at 91.3% at 28 days post-surgery. Twelve patients (7.5%) were not followed up post-discharge and 2 patients (1.3%) died prior to 28 days post-surgery.

4.3. Rationale for the trial

The routine maintenance of serum [K+] ≥ 4.5 mEq/L is a costly practice of unproven efficacy that is unpleasant and may be hazardous for patients. We shall address this issue, performing the first appropriately powered non-inferiority multicentre randomised trial of potassium supplementation. The findings will have important consequences for patients and clinicians, regardless of whether or not potassium supplementation is found to be non-inferior for the prevention of AF after cardiac surgery. A survey of practice patterns in Europe and North America suggests that there is genuine equipoise, with 67% of caregivers practising in Europe reporting that their institution has a protocol for maintaining high-normal serum potassium levels after cardiac surgery[12, 13]. So one in three do not.

The Tight K Trial will set out to test the hypothesis that AF will be no more common after coronary artery bypass graft (CABG) surgery when serum potassium levels are maintained ≥3.6 mEq/L as when they are maintained ≥4.5 mEq/L.

5. Aim and Objectives

5.1. Aim

The aim of the Tight K Trial is to determine whether maintaining serum potassium levels at ≥3.6 mEq/L is non-inferior to normal treatment (≥4.5 mEq/L) on the occurrence of new onset AF dysrhythmia post-surgery in patients undergoing CABG surgery.

5.2. Hypothesis

AF will be no more common after CABG surgery as when serum potassium levels are maintained ≥3.6 mEq/L than when they are maintained ≥4.5 mEq/L.

5.3. Objectives

5.3.1. Primary objective

- Assess whether new onset AF is just as prevalent in the first 120 hours after isolated CABG surgery, when serum $[K^+]$ is maintained ≥4.5 mEq/L, as when concentrations ≥3.6 mEq/L are targeted

5.3.2. Secondary objectives

- Estimate the AF burden on recovering CABG surgery patients
- Estimate the cost of delivering potassium interventions

6. Trial setting and design

6.1. Setting

6.1.1. Site requirements

- Perform CABG surgeries on site
- Compliance with all responsibilities as stated in the Tight K Statement of Activities
- Compliance with all requirements of the trial protocol, including the trial treatment and follow-up schedules
- Compliance with the Research Governance Framework for Health and Social Care and International Council for Harmonisation Guidelines on Good Clinical Practice (ICH-GCP)

6.1.2. Site and PI responsibilities

- Identify at least one local Principal Investigator (PI)
- Agree to incorporate the Tight K Trial into routine post-surgical and critical care clinical practice
- Adherence with the most recent approved version of the trial protocol
- Ensure training of relevant site staff in accordance with the trial protocol and ICH-GCP requirements
- Agree to randomise all eligible patients and maintain a screening log
- Agree to adhere to individual patient randomisation allocations
- Agree to timely data collection, entry and validation
- Agree to prompt notification of all adverse events

6.1.3. Site initiation and activation

The following must be in place before a site can be activated for recruitment:

- completed site initiation visit
- all relevant institutional approvals (e.g. local confirmation of capacity and capability)
- fully signed Tight K Statement of Activities
- completed Delegation Log and Training Logs

Once the London School of Hygiene & Tropical Medicine (LSHTM) Clinical Trials Unit (CTU) have confirmed that all necessary documentation is in place, a site activation email will be issued to the PI, at which point, the site may start to screen for eligible patients.

All local staff (i.e. PIs, local investigators, research teams) involved in the conduct of the trial must be listed and signed off on the Delegation Log, once trained to carry out their delegated duties. The Delegation Log should be copied and sent to the Tight K Trial Team at the LSHTM CTU whenever changes are made

6.2. Trial design

The Tight K Trial is a pragmatic, multicentre, non-inferiority randomised clinical trial (RCT).

6.2.1. Primary outcome

The presence of new onset AF until hour 120 after surgery or discharge from hospital, whichever is sooner.

6.2.1.1. Definition of AF

Atrial fibrillation will be defined as an episode of AF lasting ≥30 seconds that is both clinically detected and electrocardiographically confirmed (on either a 12-lead electrocardiogram (ECG), telemetry or Holter monitoring[24]).

AF episodes lasting <30 seconds will not be recorded as AF.

6.2.2. Secondary outcomes

- Prevalence of all AF, including those identified on holter monitors
- Prevalence of all other non-AF dysrhythmias, defined using standard diagnostic criteria
- In-patient mortality
- 6-month mortality
- Critical care length of stay
- Hospital length of stay
- Costs relating to potassium therapy
- Quality of life at 6 months

7. Selection and withdrawal of participants

7.1. Inclusion criteria

1. Undergoing isolated CABG surgery

7.2. Exclusion criteria

1. Age less than 18 years
2. Previous AF
3. On-going infection or sepsis at the time of recruitment
4. Pre-operative high-degree atrioventricular (AV) block
5. Pre-operative serum [K^+] greater than 5.5 mEq/L
6. Current or previous use of medication for the purposes of cardiac rhythm management
7. Dialysis-dependent end-stage renal failure
8. Concurrent patient involvement in another clinical study assessing cardiac rhythm post-operative interventions
9. Unable to provide informed consent

7.3. Co-enrolment

Co-enrolment with observational studies is permitted in Tight K. Co-enrolment with interventional trials will be assess by the Trial Management Group (TMG) on a case-by-case basis. The lone exception are trials assessing cardiac rhythm post-operative interventions, in which co-enrolment is prohibited.

7.4. Withdrawal of participants

7.4.1. Criteria for withdrawal from the trial

A participant may decide to withdraw from the trial at any time without prejudice to their future care.

7.4.2. Withdrawing from the trial treatment

Participants who withdraw from the trial treatment will be treated according to standard clinical care. They will continue to be followed up by the research team unless otherwise indicated by the participant.

7.4.3. Withdrawing from the trial

Participants who withdraw from the trial while in hospital will be treated according to standard clinical care. Participants who withdraw from the trial after discharge from hospital will be followed up as per standard clinical care by local clinical team. Participants will be encouraged to allow data that have been collected before withdrawal to be used in the analyses. However, if consent to use already collected data is also withdrawn, then these data will be discarded. There will be no further follow-up from the research team.

7.4.4. Reporting withdrawal

The LSHTM CTU should be informed by email if a participant has withdrawn from the trial or the trial treatment. For the former, a withdrawal form will be completed on the trial electronic case report form (eCRF).

8. Trial procedures

8.1. Screening

Staff at the participating sites will identify patients who are scheduled to have a CABG procedure from hospital waiting lists. If patients are having an isolated CABG procedure, then their notes will be reviewed to confirm that they are eligible to participate. Research staff will approach patients at their scheduled pre-assessment appointment or prior to their scheduled hospital appointment via post, telephone or email to discuss the study.

8.1.1. Screening log

Sites will complete a screening log for all patients screened for the trial. This will included patients who are randomised, who met one or more of the exclusion criteria and who were eligible but not randomised.

Anonymised screening logs will be sent monthly to the LSHTM CTU.

8.2. Informed consent procedure

Patients will be given a copy of the patient information sheet (PIS) at a pre-operative hospital appointment prior to their planned surgery date. At this appointment, the PI or another delegated member of the research team will discuss the study further and answer any questions the patient may have.

If patients are willing to take part, they can consent at the pre-operative appointment or when they are admitted to hospital for their surgery. However, it is recommended that patients are allowed 24 hours to consider whether or not to take part in the study in order for them to make an informed decision. Written consent will be obtained on a consent form. A baseline health questionnaire, the EuroQol EQ-5D 5-level questionnaire (EQ-5D-5L), will be completed by the patient at this time.

8.3. Randomisation

Patients who have consented to take part will be allocated using an online randomisation system to receive either ‘tight’ or ‘relaxed’ potassium control. Where possible, randomisation should occur on the day of the CABG surgery.

Treatment allocation will be random and in a 1:1 ratio between the two groups. The randomisation allocation sequence will be computer generated using randomly permuted blocks of varying size and stratified by site.

8.3.1. Intervention arm

Those randomised to the 'Relaxed' Group will receive K^+ supplementation only if their serum [K^+] drops below or equals 3.6mEq/L.

8.3.2. Control arm

Patients randomised to the 'Tight' group will receive K^+ supplementation if their serum [K^+] falls below 4.5 mEq/L (current practice).

8.3.3. Blinding

Blinding patients and clinical staff to the treatment allocation is not possible. The analysis of the outcomes and holter monitor data will be carried out blind to the treatment allocation.

8.4. Trial treatment

The trial treatment will start when participants are admitted to the ICU or another post-operative care facility after their surgery. The participant will undergo regular blood investigations, as per current practice. The frequency of [K+] monitoring will be according to local protocols, clinician/nursing staff preference and clinical need. All other treatments will be given according to standard clinical care and clinician's preference.

The administration route used for all potassium replacement will be prescribed according to clinician preference and given according to existing standardised protocols. This may include intravenous (IV) or oral potassium formulation, administration of potassium-rich nasogastric feeding regimens, recommending the consumption of potassium-rich foods or avoidance of potassium losing drugs.

The use of IV magnesium, beta-blockers and anti-dysrhythmic agents will be as per current practice in both groups.

The trial treatment period will end 120 hours (5 days) after initial admission to ICU/post-operative care facility, discharge from hospital, or with occurrence of a clinically-identified episode of AF (see Section 8.4.1) – whichever occurs first.

8.4.1. Patients with AF

Once a participant has a trial-defined period of AF (see Section 6.2.6.1), there will be no restriction on potassium supplementation and the participant should be treated according to current practice. However, holter monitoring and data collection should continue until the end of the 120 hours or discharge from hospital.

8.5. Holter monitoring

In addition to the usual care, participants will also be asked to wear an external heart rhythm monitor for up to 120 hours (5 days) following their CABG surgery. This will monitor their heart rhythm for any irregular heart rhythms conditions such as AF.

Holter monitor data will be reviewed by a core laboratory group based at Wythenshawe Hospital, Manchester University NHS Foundation Trust. Pseudnonymised patient data will be analysed blinded to participant allocation, with some delay to the acute episode of care

and will only be reviewed post-hoc. If the analysis flags up any malignant dysrhythmias, then these will be fed back to the participant's primary care team, as and when that information becomes available. It is important to note that in clinical practice, holter monitor data are diagnostic, but not prognostic, and the way they are used here in the context of a research trial is no different.

8.6. Follow-up

All participants will be followed up 6 months (+/- 1 month) after their surgery. Follow-up will occur either in person, via a telephone call, email or post. An EQ-5D-5L questionnaire will be completed by the patient at this time. Participants will be asked to provide information about further incidences of AF and other heart rhythm problems, and stroke after their hospital discharge, if known.

Participants will also be followed up remotely via NHS Digital for any hospitalisations for AF between discharge and 6 months post-surgery.

8.7. End of trial

The end of the trial is defined as last participant, last follow-up.

9. Data collection

9.1. Trial treatment period

The trial treatment period commences after surgery when the participant is admitted to ICU or another post-operative care facility after their surgery. The participant's inclusion into the trial and randomised allocation must be clear upon admission to ICU or another post-operative care facility.

Period 1 (0–24 hours post-admission to ICU)
Period 2 (24-48 hours post-admission to ICU)
Period 3 (48-72 hours post-admission to ICU)
Period 4 (72-96 hours post-admission to ICU)
Period 5 (96-120 hours post-admission to ICU)
Follow-up (6 months (+/- 1 month) post-CABG surgery)

9.2. Trial Procedures Table

	Before Surgery	Day of Surgery	ICU Stay (commences on admission to ICU)					Discharge	Follow-up
			Period 1	Period 2	Period 3	Period 4	Period 5		
Review of eligibility criteria	X								
Consent	X								
Baseline	X								
Randomisation		X							
Trial treatment			X	X	X	X	X		
Holter Monitoring			X	X	X	X	X		
Clinical events			X	X	X	X	X	X	X
Safety monitoring			X	X	X	X	X	X	X
EQ-5D-5L	X								X

9.3. Data collection

Data collected for all participants will include measurements of daily serum electrolytes / renal function and collation of adverse events attributed to K^+ replacement, including gastrointestinal symptoms from oral K^+ replacement. Medication at hospital discharge will be collated, including whether anticoagulation is commenced for atrial fibrillation. Additional staff time for delivering the intervention will be recorded on site visits and will be informed by expert clinical view.

Detailed information will be collected on the resource use associated with delivering each protocol, including the total number of replacement K^+ interventions and the number of tests for monitoring potassium levels.

The perceived clinical impact (symptomatic/asymptomatic) of a dysrhythmia will be noted by the clinical staff. Participants in either group who experience AF (as defined in Section 6.2.6.1) will be deemed to have met the primary end point.

9.4. Trial procedures

9.4.1. Before surgery

- Consent
- Quality of life questionnaire (EQ-5D-5L)

9.4.2. Baseline

- Age
- Gender
- Ethnic origin
- Cardiac medication and indication (including beta-blockers, calcium channel blockers, ACE-inhibitors, all blockers, aldosterone antagonists, anticoagulation)
- Medical history: family history of dysrhythmia (and details), chronic obstructive pulmonary disease/lung disease, diabetes mellitus (and type), hypertension, myocardial infarction, chronic kidney disease, transient ischaemic attack or stroke / cerebrovascular accident
- Imaging data: Left ventricular ejection fraction / left atrial size, mitral regurgitation or stenosis (defined as moderate or worse) and estimated pulmonary artery systolic pressure
- HAS-BLED and CHADSVASC scores will be calculated

9.4.3. Period 1-5

- Potassium blood readings (measured as per clinician preference and local pathways)
- Potassium administration (dose and route)
- Clinically significant pacing modes
- Restenotomy
- Clinical events
- Safety monitoring

9.4.4. Discharge (from ICU and hospital)

- ICU length of stay
- Hospital length of stay
- Medications
- Duration of central venous lines left in situ
- Clinical events
- Safety monitoring

9.4.5. Follow-up

- Quality of life questionnaire (EQ-5D-5L)
- Clinical events
- Safety monitoring

9.5. Compliance and loss to follow-up

9.5.1. Loss to follow-up

The majority of participants remain in hospital for 5 days after their CABG surgery, so loss to follow up over that period is unlikely.

The participants will subsequently be followed up at 6 months following CABG surgery. Participants will be given an ID card to remind them they are taking part in the study and to optimise event reporting during follow-up to the trial team.

9.5.2. Compliance

Given that a large number of different health providers care for post-operative patients in different hospital locations, it is conceivable that protocol violations will occur. These violations may either result from a patient from the 'Relaxed' group being treated as if they are in the 'Tight' Group, or visa-versa.

It is more likely that protocol violations will occur once the patient leaves the ICU and arrives on the post-operative step down ward. These wards have lower staff-to-patient ratios and a higher turnover of staff members. It is critical for the success of the trial that junior doctors and nursing staff in all the post-operative areas where patients may spend time are informed about the trial's goals and protocols. Patients, as they are not blinded, can also play a role in reminding staff which group they have been allocated to. The trial is not blinded to caregivers on the ICU/surgical ward so there is always a risk of concomitant treatment bias, but training will be put in place to minimise this.

9.6. Data Handling and Record Keeping

Data will be entered onto an online database and stored securely on Rackspace servers; http://www.rackspace.co.uk and managed by Sealed Envelope™. Data will be kept for 20 years following completion of the trial.

Pseudonymised patient data will be stored on non-networked PCs at the Core Lab at Wythenshawe Hospital, Manchester University NHS Foundation Trust, with regular external code-locked USBs (with or without secure cloud back-up).

The data controller for the trial is the Chief Investigator (Barts Health NHS Trust are the data controller's organisation) and the data processor is LSHTM and Wythenshawe Hospital.

Patient data will be kept confidential and managed in accordance with the Data Protection Act (2018), NHS Caldecott principles, the Research Governance Framework for Health and Social Care, and the conditions of Research Ethics Committee Approval.

10. Monitoring and Audits

The conduct of the trial will be supervised by trained staff from the LSHTM CTU. The trial will be monitored on a regular basis using central statistical monitoring. Full details will be available in the monitoring standard operating procedure (SOP) and the trial will be monitored according to this agreed plan.

Local investigators shall ensure that all trial data are available for trial-related monitoring, and sponsor and regulatory authority audits. Sponsor also holds the right to monitor or audit the study.

11. Safety monitoring

11.1. Definition

Unexpected events that have not been defined as outcomes (see Section 6.2.1, 6.2.2), expected complications of potassium supplementation, expected complications of CABG surgery or expected complications of usual clinical care (see Section 11.2) should be reported as either a serious adverse event (SAE) or non-serious adverse event (NSAE), depending on their severity. Safety reporting for each patient should commence from time of randomisation to completion of follow-up at 6 months after the CABG surgery.

11.2. Expected adverse events

- Skin irritation from ECG electrodes
- Hyperkalaemia ($[K^+]$ ≥5.5 mEq/L)
- Line site complications (phlebitis, infection etc.)
- Nausea
- Constipation, vomiting (due to receiving oral potassium supplementation)
- Myocardial infarction
- Stroke
- Renal failure requiring dialysis
- Renal impairment not requiring dialysis
- Wound infection (sternum or donor site)
- Return to theatre for bleeding
- Prolonged mechanical support
- Post-op delirium
- Non-cardiac chest pain
- Heart failure
- Pleural effusion
- Chest drain insertion
- Pericardial effusion
- Chest infection (pneumonia)
- Lung atelectasis
- Pneumothorax
- Shortness of breath caused by any of the above

- Blood transfusion
- Pericarditis
- Ulnar nerve paraesthesia
- Heart block requiring pacemaker
- Urinary tract infection
- Suprapubic catheter
- Urinary retention
- Intra-aortic balloon pump insertion

11.3. Unexpected Serious Adverse Events

Any untoward medical occurrence/effect that:

1. Results in death
2. Is life-threatening*
3. Requires hospitalisation or prolongation of existing hospitalisation
4. Results in persistent or significant disability or incapacity
5. Consists of a congenital anomaly or birth defect
6. Is otherwise considered medically significant by the investigator

*Life-threatening, in the definition of a SAE, refers to an event in which the patient was at risk of death at the time of event. It does not refer to an event that hypothetically might have caused death if it were more severe.

Unexpected SAEs should be reported to the CTU within 7 days. The report should include an assessment of seriousness and causality (see Section 11.5.2) by the Principal Investigator, or a member of staff delegated this task, at each site.

The Chief Investigator will be responsible for the prompt notification of findings that could adversely affect the health of patients or impact on the conduct of the trial. Notification of confirmed **unexpected** and **related** SAEs will be to the Sponsor, the Research Ethics Committee (REC) and the Data and Safety Monitoring Committee (DSMC).

11.4. Unexpected Non-Serious Adverse Events

Unexpected NSAEs should be evaluated by the PI or a member of staff delegated this task. This should include an assessment of causality (see Section 11.5.2) and intensity (see Section 11.5.1) and reports made within 14 days. The CTU will keep detailed records of all unexpected adverse events reported. Reports will be reviewed by the Chief Investigator to consider intensity, causality and expectedness. As appropriate, these will be reported to the Sponsor, the DSMC and the REC.

11.5. Reporting unexpected adverse events

Investigators will make their reports of all unexpected adverse events, whether serious or not, to the CTU at LSHTM.

11.5.1. Assessment of intensity

Mild: The patient is aware of the event or symptom, but the event or symptom is easily tolerated.

Moderate: The patient experiences sufficient discomfort to interfere with or reduce his or her usual level of activity.
Severe: Significant impairment of functioning; the patient is unable to carry out usual activities and/or the patient's life is at risk from the event.

11.5.2. Assessment of causality

Probable: A causal relationship is clinically / biologically highly plausible and there is a plausible time sequence between onset of the adverse event and the RIC procedure.
Possible: A causal relationship is clinically / biologically plausible and there is a plausible time sequence between onset of the adverse event and the RIC procedure.
Unlikely: A causal relationship is improbable and another documented cause of the adverse event is most plausible.
Unrelated: A causal relationship can definitely be excluded and another documented cause of the adverse event is most plausible.

11.6. Urgent Safety Measures

The Chief Investigator may take urgent safety measures to ensure the safety and protection of the clinical trial patients from any immediate hazard to their health and safety. The measures should be taken immediately. In this instance, the approval of the REC prior to implementing these safety measures is not required. However, it is the responsibility of the Chief Investigator to inform the Sponsor and REC (via telephone) of this event immediately.

The Chief Investigator has an obligation to inform the REC in writing within 3 days, in the form of a substantial amendment. The Sponsor (Joint Research Management Office [JRMO]) must be sent a copy of the correspondence.

11.7. Annual Safety Reporting

The Chief Investigator will send an Annual Progress Report to the main REC using their template (the anniversary date is the date on the REC "favourable opinion" letter from the REC) and to the Sponsor.

11.8. Overview of the Safety Reporting responsibilities

The Chief Investigator has the overall safety oversight responsibility. The Chief Investigator has a duty to ensure that safety monitoring and reporting is conducted in accordance with the Sponsor's requirements.

12. Statistical Considerations

12.1. Power calculations and sample size determination

1684 participants are to be recruited from 15-25 centres allocated in a ratio of 1:1.

The sample-size calculation is based on a prevalence of new onset AF in the tight potassium control arm of 35%, which is at the lower end of the published figures and supported by our pilot data that showed an overall prevalence of 36.8% (95% confidence interval [CI] 29.1 to 44.9).

The co-applicants (from diverse backgrounds in cardiothoracic surgery, cardiothoracic intensive care, cardiology and clinical trial management) reached consensus that that a clinically relevant non-inferiority margin is 10%. If there is a true difference in favour of tight potassium control of 2%, then 1514 participants are required to be 90% certain that the upper limit of a one-sided 97.5% CI (or equivalently a 95% two-sided CI) will exclude a difference in favour of tight potassium control of more than 10%. Allowing for a 10% loss to follow-up means we need to recruit 1684 participants.

12.1.1. Tight K Australia

The Tight K Trial (UK) is a collaboration with a partner trial in Australia (also called Tight K), which will recruit 550 participants. The UK trial is sufficiently powered on its own. The data from both trials will form a prospective meta-analysis, providing further power and aiding generalisability.

12.2. Trial statistician

Statistical analysis will be coordinated from the CTU at LSHTM.

12.3. Statistical analysis

Statistical analysis will be carried out blind to treatment allocation.

12.3.1. Summary of baseline data and flow of participants

Baseline characteristics of enrolled participants will be summarised by treatment arm. Descriptive statistics for continuous variables will include mean, standard deviation, median, range and number of observations. Categorical variables will be summarised as counts and proportions. Screening, enrolment, reasons for non-enrolment, randomisation and loss to follow-up will be detailed in a CONSORT flowchart.

12.3.2. Primary and secondary outcome analyses

As this is a non-inferiority trial, the primary and secondary outcome analyses will be carried out on the per-protocol population. An intention-to-treat (ITT) analysis will also be carried out. Analysis will follow a pre-specified analysis plan approved by the senior statistician and Chief Investigator prior to unblinding the study database. The criterion for statistical significance at the final analysis will be $P<0.05$. The rate of loss to follow-up will be reported. We will report risk ratios for prevalence of new onset AF hour 120 after surgery or discharge from hospital (primary outcome) and for inpatient and six month mortality after enrolment

(secondary outcomes) for intervention versus control with associated 95% CI. Secondary outcomes: length of ICU and length of hospital stay, will be analysed using Kaplan-Meier plots and the hazard ratios with accompanying 95% CI calculated using Cox proportional hazards regression. All other secondary outcomes will be analysed using appropriate regression models accounting for the nature of the distribution of the outcome and results will be presented as appropriate effect sizes with a measure of precision (95% CI). Both unadjusted analyses and analyses adjusted for the stratification factor will be carried out. Additional exploratory analyses will control for any baseline measures that appear to be imbalanced between arms.

13. Ethics

13.1. Declaration of Helsinki and Good Clinical Practice

The study will conform to the spirit and the letter of the declaration of Helsinki, and in accordance with the Barts Health and ICH-GCP. The study will be carried out in accordance with the ethical principles in the Research Governance Framework for Health and Social Care, Second Edition, 2005 and its subsequent amendments as applicable and applicable legal and regulatory requirements.

13.2. Ethical committee review

Health Research Authority (HRA) Research Ethics Committee *TO BE ADDED* have reviewed and approved the trial. The REC number is *TO BE ADDED*. Copies of the letters of approval will be filed in the trial site files at each centre.

13.3. Confidentiality Advisory Group

Written consent will not be available for delegated members of the research team to access hospital notes for patients undergoing CABG surgery to screen them for eligibility. Permission has been granted by the Confidentiality Advisory Group (CAG) to allow notes to be screened and for staff to contact patients prior to a clinic appointment to inform them about the trial. The CAG reference number *TO BE ADDED*.

14. Management and oversight

14.1. Trial Management Group (TMG)

Prof Ben O'Brien (St Bartholomew's Hospital)
Prof Julie Sanders (St Bartholomew's Hospital)
Mr Neil Roberts (St Bartholomew's Hospital)
Dr Niall Campbell (Wythenshawe Hospital)
Prof Hugh Montgomery (University College London)
Ms Trudie Lobban (Arrhythmia Alliance)
Prof Diana Elbourne (LSHTM)
Prof Liz Allen (LSHTM)
Dr Zia Sadique (LSHTM)
Ms Laura Van Dyck (LSHTM)
Ms Ruth Canter (LSHTM)
Mr Richard Evans (LSHTM)

14.2. Trial Steering Committee (TSC)

Prof Monty Mythen (University College London) – Chair
Mr Jatin Desai (King's College Hospital, retired) – Independent
Dr Matthew Lovell (Royal Devon and Exeter Hospital) – Independent
Dr Kurt Rützler (Cleveland Clinic, USA) – Independent
Mr Steve Stevenson (Patient representative) – Independent
Mr Richard Duncker (Patient representative) – Independent
Ms Beatrice Moloce (Royal Brompton Hospital) – Non-independent
Dr Nick Barrett (St Thomas' Hospital) – Non-independent
Prof Ben O'Brien (St Bartholomew's Hospital) – Chief Investigator
Prof Diana Elbourne (LSHTM) – Co-investigator

14.3. Data Safety and Monitoring Committee (DSMC)

Dr Philip Jones (University of Western Ontario, Canada) – Chair
Dr Ly-Mee Yu (University of Oxford)
Prof Thomas Walther (University Hospital in Frankfurt (Main), Germany)

15. Finance and Funding

This trial is funded by the BHF.

16. Indemnity

16.1. Sponsorship

This trial is sponsored by Barts Health NHS Trust.

16.2. Insurance

All recruiting centres will be covered by NHS indemnity for negligent harm providing researchers hold a contract of employment with the NHS, including honorary contracts held

by academic staff. Medical co-investigators will also be covered by their own medical defence insurance for non-negligent harm.

17. Dissemination of Research Findings

It is our intention to disseminate the results of the trial as widely as possible, including to the patients who participated. This is likely to be through a publication in a peer reviewed journal. Publications will follow the CONSORT guidelines. Authorship will follow international guidelines.

18. References

1. Maisel WH, Rawn JD, Stevenson WG. Atrial fibrillation after cardiac surgery. Annals of internal medicine. 2001 Dec 18;135(12):1061-73
2. Mathew JP, Fontes ML, Tudor IC, Ramsay J, Duke P, Mazer CD, et al. A multicenter risk index for atrial fibrillation after cardiac surgery. Jama. 2004 Apr 14;291(14):1720-9
3. Helgadottir S, Sigurdsson MI, Ingvarsdottir IL, Arnar DO, Gudbjartsson T. Atrial fibrillation following cardiac surgery: risk analysis and long-term survival. Journal of cardiothoracic surgery. 2012 Sep 19;7:87
4. Villareal RP, Hariharan R, Liu BC, Kar B, Lee VV, Elayda M, et al. Postoperative atrial fibrillation and mortality after coronary artery bypass surgery. J Am Coll Cardiol. 2004 Mar 3;43(5):742-8
5. Mariscalco G, Klersy C, Zanobini M, Banach M, Ferrarese S, Borsani P, et al. Atrial fibrillation after isolated coronary surgery affects late survival. Circulation. 2008 Oct 14;118(16):1612-8
6. El-Chami MF, Kilgo P, Thourani V, Lattouf OM, Delurgio DB, Guyton RA, et al. New-onset atrial fibrillation predicts long-term mortality after coronary artery bypass graft. J Am Coll Cardiol. 2010 Mar 30;55(13):1370-6
7. Sanders J, Keogh BE, Van der Meulen J, Browne JP, Treasure T, Mythen MG, et al. The development of a postoperative morbidity score to assess total morbidity burden after cardiac surgery. Journal of clinical epidemiology. 2012;65(4):423-33
8. Aranki SF, Shaw DP, Adams DH, Rizzo RJ, Couper GS, VanderVliet M, et al. Predictors of atrial fibrillation after coronary artery surgery. Current trends and impact on hospital resources. Circulation. 1996 Aug 1;94(3):390-7
9. Zimmer J, Pezzullo J, Choucair W, Southard J, Kokkinos P, Karasik P, et al. Meta-analysis of antiarrhythmic therapy in the prevention of postoperative atrial fibrillation and the effect on hospital length of stay, costs, cerebrovascular accidents, and mortality in patients undergoing cardiac surgery. The American journal of cardiology. 2003;91(9):1137-40
10. Borzak S, Tisdale JE, Amin NB, Goldberg AD, Frank D, Padhi ID, et al. Atrial fibrillation after bypass surgery: does the arrhythmia or the characteristics of the patients prolong hospital stay? Chest. 1998 Jun;113(6):1489-91
11. Schnabel RB, Sullivan LM, Levy D, Pencina MJ, Massaro JM, D'Agostino RB, Sr., et al. Development of a risk score for atrial fibrillation (Framingham Heart Study): a community-based cohort study. Lancet (London, England). 2009 Feb 28;373(9665):739-45
12. O'Brien B, Burrage PS, Ngai JY, Prutkin JM, Huang CC, Xu X, et al. Society of Cardiovascular Anesthesiologists/European Association of Cardiothoracic Anaesthetists Practice Advisory for the Management of Perioperative Atrial Fibrillation in Patients Undergoing Cardiac Surgery. Journal of cardiothoracic and vascular anesthesia. 2019 Jan;33(1):12-26
13. Muehlschlegel JD, Burrage PS, Ngai JY, Prutkin JM, Huang CC, Xu X, et al. Society of Cardiovascular Anesthesiologists/European Association of Cardiothoracic Anaesthetists Practice Advisory for the Management of Perioperative Atrial Fibrillation in Patients Undergoing Cardiac Surgery. Anesthesia and analgesia. 2019 Jan;128(1):33-42

14. Podrid PJ. Potassium and ventricular arrhythmias. Am J Cardiol. 1990 Mar 6;65(10):33E-44E; discussion 52E
15. Polderman KH, Girbes AR. Severe electrolyte disorders following cardiac surgery: a prospective controlled observational study. Crit Care. 2004 Dec;8(6):R459-66
16. Krijthe BP, Heeringa J, Kors JA, Hofman A, Franco OH, Witteman JC, et al. Serum potassium levels and the risk of atrial fibrillation: the Rotterdam Study. International journal of cardiology. 2013 Oct 15;168(6):5411-5
17. Dunning J, Treasure T, Versteegh M, Nashef SA. Guidelines on the prevention and management of de novo atrial fibrillation after cardiac and thoracic surgery. European journal of cardio-thoracic surgery : official journal of the European Association for Cardio-thoracic Surgery. 2006 Dec;30(6):852-72
18. Arsenault KA, Yusuf AM, Crystal E, Healey JS, Morillo CA, Nair GM, et al. Interventions for preventing post-operative atrial fibrillation in patients undergoing heart surgery. Cochrane Database of Systematic Reviews [Internet]. 2013; (1). Available from: http://onlinelibrary.wiley.com/doi/10.1002/14651858.CD003611.pub3/abstract.
19. Weiner ID, Wingo CS. Hypokalemia--consequences, causes, and correction. Journal of the American Society of Nephrology : JASN. 1997 Jul;8(7):1179-88
20. Pittet D, Tarara D, Wenzel RP. Nosocomial bloodstream infection in critically ill patients. Excess length of stay, extra costs, and attributable mortality. Jama. 1994 May 25;271(20):1598-601
21. Norris W, Kunzelman KS, Bussell S, Rohweder L, Cochran RP. Potassium Supplementation, Diet vs Pills*. Chest. 2004 February 1, 2004;125(2):404-9
22. Cohn JN, Kowey PR, Whelton PK, Prisant LM. New Guidelines for Potassium Replacement in Clinical Practice: A Contemporary Review by the National Council on Potassium in Clinical Practice. Arch Int Med2000. p. 2429-36.
23. Pilot Study for the Tight K Study (Tight K) [14/02/2019]. Available from: https://clinicaltrials.gov/ct2/show/NCT03195647.
24. Calkins H, Kuck KH, Cappato R, Brugada J, Camm AJ, Chen SA, et al. 2012 HRS/EHRA/ECAS expert consensus statement on catheter and surgical ablation of atrial fibrillation: recommendations for patient selection, procedural techniques, patient management and follow-up, definitions, endpoints, and research trial design: a report of the Heart Rhythm Society (HRS) Task Force on Catheter and Surgical Ablation of Atrial Fibrillation. Developed in partnership with the European Heart Rhythm Association (EHRA), a registered branch of the European Society of Cardiology (ESC) and the European Cardiac Arrhythmia Society (ECAS); and in collaboration with the American College of Cardiology (ACC), American Heart Association (AHA), the Asia Pacific Heart Rhythm Society (APHRS), and the Society of Thoracic Surgeons (STS). Endorsed by the governing bodies of the American College of Cardiology Foundation, the American Heart Association, the European Cardiac Arrhythmia Society, the European Heart Rhythm Association, the Society of Thoracic Surgeons, the Asia Pacific Heart Rhythm Society, and the Heart Rhythm Society. Heart rhythm : the official journal of the Heart Rhythm Society. 2012 Apr;9(4):632-96 e21
